# Human cellular model systems of β-thalassemia enable in-depth analysis of disease phenotype

**DOI:** 10.1038/s41467-023-41961-9

**Published:** 2023-10-06

**Authors:** Deborah E. Daniels, Ivan Ferrer-Vicens, Joseph Hawksworth, Tatyana N. Andrienko, Elizabeth M. Finnie, Natalie S. Bretherton, Daniel C. J. Ferguson, A. Sofia. F. Oliveira, Jenn-Yeu A. Szeto, Marieangela C. Wilson, John N. Brewin, Jan Frayne

**Affiliations:** 1 School of Biochemistry, University of Bristol, Bristol, BS8 1TD UK; 2 Haematology Department, King’s college Hospital NHS Foundation, London, SE5 9RS UK; 3 Red Cell Biology Group, Kings College London, London, SE5 9NU UK

**Keywords:** Anaemia, Mechanisms of disease, Experimental models of disease

## Abstract

β-thalassemia is a prevalent genetic disorder causing severe anemia due to defective erythropoiesis, with few treatment options. Studying the underlying molecular defects is impeded by paucity of suitable patient material. In this study we create human disease cellular model systems for β-thalassemia by gene editing the erythroid line BEL-A, which accurately recapitulate the phenotype of patient erythroid cells. We also develop a high throughput compatible fluorometric-based assay for evaluating severity of disease phenotype and utilize the assay to demonstrate that the lines respond appropriately to verified reagents. We next use the lines to perform extensive analysis of the altered molecular mechanisms in β-thalassemia erythroid cells, revealing upregulation of a wide range of biological pathways and processes along with potential novel targets for therapeutic investigation. Overall, the lines provide a sustainable supply of disease cells as research tools for identifying therapeutic targets and as screening platforms for new drugs and reagents.

## Abstract (web summary)

β-thalassemia is a prevalent genetic disorder causing severe anemia, with study of the underlying molecular defects impeded by paucity of suitable patient material. Here, the authors show that cellular model systems of βthalassemia can be used to identify new therapeutic targets and as screening platforms for new drugs and reagents.

## Introduction

β-thalassemia syndromes are a heterogeneous range of anemias and a major source of morbidity, mortality, and substantial financial burden globally. The disease is characterized by variable reduced (β^+^) or absent (β^0^) β-globin chain synthesis, with more than 300 different mutations in and around the β globin gene identified^1^. Mutations resulting in β^0^-thalassemia cause a severe chronic anemia, whereas that resulting from β^+^ varies from clinically benign to severe.

Reduction in β-globin results in excess α-globin, forming insoluble aggregates which undergo auto-oxidation leading to increased reactive oxygen species (ROS) and a range of intracellular downstream events that result in ineffective erythropoiesis (IE), the hallmark and primary cause of β-thalassemia disease pathophysiology. IE is manifest as increased expansion of erythroid progenitors due to increased EPO production in vivo, but with a maturation block at the polychromatic stage of differentiation^2,3^ with increased apoptosis (reviewed by Ribeil et al. ^4^), and thus impaired erythropoiesis. However, the underlying molecular mechanisms that cause IE are not fully understood.

In recent years there has been much renewed interest in studying β-thalassemia, however there are still few treatment options. The mainstay is blood transfusion, but this has associated complications of alloimmunisation and iron-mediated potentially fatal organ damage, requiring life-long iron-chelation therapy.

Non-curative approaches have aimed to correct globin imbalance by promoting γ-globin synthesis, such as with hydroxyurea^5^, but responses are inconsistent^6–8^. More recently, luspatercept has been shown to increase hemoglobin values, but modestly by 1–2 g/dl in non-transfusion dependent thalassemia patients (NTDT). However, only 20% of transfusion-dependent (TDT) patients showed a reduction of blood transfusion by more than a third^9^. The only definitive cure is bone marrow transplant, best applicable for some young patients with a matched sibling lacking TDT^5^. Gene therapy, either using gene replacement or editing with associated bone marrow transplantation holds much promise for those lacking a matched donor^10^. However, efficacy and safety issues are still concerns and given the complexity and high cost of these approaches, they are unlikely to scale to the massive global unmet need for β-thalassemia therapeutics, particularly in low to middle income countries^11^. Thus, new cost-effective treatment strategies and drug targets are desperately required to deliver optimal therapies to the greatest numbers of people.

However, studying the molecular defects underlying the β-thalassemia phenotype, and identifying drug targets, is severely impeded by paucity of suitable material from patients, and lack of suitable cell lines. Although erythroid cells can be generated in vitro from peripheral blood stem cells, the approach is severely limited by the restricted expansion potential of the cells^12^ and thus number of erythroid cells generated, with repeat collections required, a particularly unsuitable approach for anemic patients. Thus, currently much of our understanding is based on mouse models which are also used to investigate and evaluate the effect of drugs. However, fundamental differences between mouse and human erythropoiesis are becoming increasingly apparent^13,14^. Therefore, new cellular models for β-thalassemia are essential to enable in-depth investigation of underlying molecular mechanisms, to aid identification of new therapeutic targets and as screening platforms for the effect and efficacy of potential new drugs and reagents.

In this study we created and validated human disease erythroid cell lines for β-thalassemia. Homozygous (*HBB*^-/-^) and heterozygous (*HBB*^+/−^) *HBB* gene knockout mutations, and the prevalent β-thalassemia mutations CD41/42 -TTCT and IVS-1-1 G → T, were introduced into the erythroid cell line BEL-A, which undergoes normal erythropoiesis and expresses a normal adult globin profile^15^, via CRISPR genome editing. All disease lines accurately recapitulate the disease phenotype of β^0^-thalassemia patient erythroid cells, whilst the *HBB*^+/−^ lines recapitulate cells of individuals with β-thalassemia trait. These lines provide a sustainable and consistent supply of disease cells as unique research tools, and as drug screening platforms. For the latter we also developed a high throughput compatible flow cytometry assay for evaluation of severity of IE. We utilized the assay to demonstrate that the *HBB*^−/−^ line responds appropriately to drugs known to increase γ-globin (hydroxyurea, pomalidomide and decitabine) and to *BCL11A* + 58 enhancer editing, with a correlating improvement to IE, validating the line for screening applications.

We also performed TMT-based comparative proteomic analysis. This confirmed the same profile of proteins previously reported dysregulated in β-thalassemia erythroid cells, providing further validation, whilst also providing more detailed insights on the altered molecular mechanisms in β-thalassemia erythroid cells. This included increased levels of antioxidant response enzymes, heat shock proteins and chaperones, and many proteins of the ubiquitin-proteasome and autophagy pathways along with proteins associated with lysosome biogenesis, together with up-regulation of respective pathways. The data also revealed upregulated pathways and increased levels of proteins for heme biosynthesis, along with associated upregulated endosome biogenesis and trafficking pathways, which may contribute to the disease phenotype. In addition, interrogation of the dataset revealed potential new therapeutic targeting opportunities.

Finally, we also describe a novel β-globin splice variant arising from the IVS-1-1 G → T mutation which is unable to form stable tetramers with α-globin, the variant instead precipitating and potentially contributing to the IE phenotype in IVS-1-1 erythroid cells.

## Results

The erythroid cell line BEL-A recapitulates normal adult erythroid cell differentiation and has a normal adult globin expression profile^15^. It is therefore an excellent founder line to introduce mutations to create cellular model systems of red blood cell diseases, and so was used in this study to create β-thalassemia disease lines.

### Generating *HBB*^+/−^ and *HBB*^−^^/−^ erythroid progenitor cell lines

**Fig. 1:**
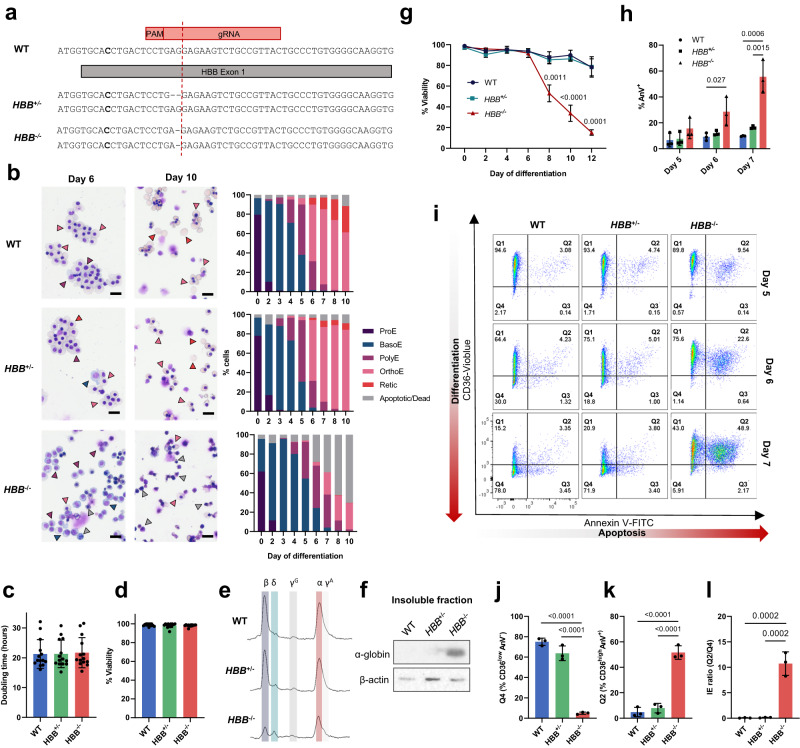
Generation and characterization of *HBB*^+/−^ and *HBB*^−/−^ erythroid progenitor cell lines. **a** Schematic of DNA sequence for *HBB*^+/−^ and *HBB*^−/−^ compared to WT BEL-A cells. **b** Representative cytospins of WT, *HBB*^+/−^ and *HBB*^−/−^ BEL-A with morphology quantification (ProE, proerythroblast; BasoE, basophilic erythroblast; PolyE, polychromatic erythroblast; OrthoE, orthochromatic erythroblast; Retic, reticulocyte). Arrowheads indicate the following cell types: blue, BasoE; purple, PolyE; pink, OrthoE; red, Retic; gray, dead/apoptotic. Scale bars 20 μm. Doubling time (**c**) and % viability (**d**) of expanding cell lines as determined by trypan blue exclusion assay, *n* = 14 independent time points. **e** RP-HPLC traces for WT, *HBB*^−/−^ and *HBB*^+/−^ BEL-A at day 6 of differentiation. Peaks are identified for β-globin (β), δ-globin (δ), ^G^γ-globin (^G^γ), α-globin (α) and ^A^γ-globin (^A^γ). **f** Representative western blot of aggregate proteins from WT, *HBB*^+/−^ and *HBB*^−/−^ BEL-A harvested at day 6 of differentiation, incubated with α-globin antibody. β-actin was used as a protein loading control. **g** Percentage viability of WT, *HBB*^−/−^ and *HBB*^+/−^ BEL-A during differentiation by trypan blue exclusion assay. **h** Percentage of Annexin V positive cells (AnV^+^) in WT, *HBB*^+/−^ and *HBB*^−/−^ BEL-A during differentiation as determined by flow cytometry. **i** Representative IE flow cytometry plots at day 5, 6 and 7 of differentiation. Quantification of Q4 CD36^low^AnV^−^ cells (**j**), Q2 CD36^high^AnV^+^ (**k**) and IE ratio (Q2/Q4) (**l**) at day 7 of differentiation. Results show mean ± SD, *n* = 3 independent experiments unless otherwise stated. Statistical significance was determined using one-way ANOVA with Tukey multiple comparison testing. Source data are provided as a Source Data file.

As β-thalassemia is caused by a wide range of mutations that inhibit or reduce the production of β-globin, in the first instance homozygous (*HBB*^−/−^) and control heterozygous (*HBB*^+/−^) β-globin knockout lines were created using CRISPR-Cas9 mediated genome editing of BEL-A. This resulted in biallelic 1 base pair and monoallelic 2 base pair deletions for the *HBB*^−/−^ and *HBB*^+/−^ clonal lines respectively (Fig. 1a), leading to frameshift and premature stop codons in exon 1 in both cases.

The resultant *HBB*^+/−^ and *HBB*^−/−^ lines had a predominantly pro-erythroblast morphology with some basophilic erythroblasts, in line with the WT BEL-A line (Fig. 1b). Doubling times and viability were invariant across the lines (Fig. 1c and d).

Analysis of globin levels by RP-HPLC showed that *HBB*^*+/*−^ cells produced similar levels of all globin subunits to WT cells whereas, as expected, β-globin was eliminated in the *HBB*^−/−^ line (Fig. 1e; see also Supplementary Fig. 1 for clarification of residual peak overlapping position of β-globin). α-globin was also reduced in the *HBB*^−/−^ cells as, in absence of β- (or γ-) globin it forms insoluble aggregates (Fig. 1f), a feature of β-thalassemia patient erythroid cells.

When differentiated, viability of the WT and *HBB*^+/-^ line remained high throughout the culture duration (Fig. 1g). In contrast the *HBB*^−/−^ line recapitulated the phenotype of β^0^-thalassemia erythroid cells^2^ with clear ineffective erythropoiesis, as demonstrated by a dramatic and significant decline in viability from day 6 of differentiation (Fig. 1g), corresponding temporally with differentiation to polychromatic erythroblasts (Fig. 1b), and with a significant increase in Annexin V (AnV) cell surface abundance (Fig. 1h), indicative of apoptosis (Fig. 1b). In line, growth curves during differentiation showed unchanged expansion in *HBB*^+/−^ and *HBB*^−/−^ lines compared to WT up until day 6, but with greater reduction in cell numbers post-day 6 in *HBB*^−/−^ line due to cell death (Supplementary Fig. 2). Similarly, kinetics of the *HBB*^+/−^ cultures were invariant to that of WT cells during differentiation (Fig. 1b). In contrast, although the majority of *HBB*^−*/*−^ cells differentiated to polychromatic erythroblasts, few differentiated further to orthochromatic erythroblasts (Fig. 1b), as found for cultured patient erythroid cells^2^.

Analysis of a second *HBB*^−/−^ clonal line (*HBB*^−/−^C2) displayed the same phenotype, with similar decrease in viability from day 6 of culture and associated significant increase in AnV levels and apoptosis at the polychromatic stage (Supplementary Fig. 3A–C).

Overall, the data confirm the *HBB*^−/−^ lines as robust cellular models of β^0^-thalassemia, providing a sustainable and consistent supply of disease cells.

### Assay for assessing severity of ineffective erythropoiesis

To evaluate drugs and reagents that reduce severity of IE, thus improving disease phenotype, the WT, *HBB*^+/−^ and *HBB*^−/−^ lines were used to establish a flow cytometric assay for IE utilizing fluorophore tagged antibodies to membrane markers. For this, the abundance of membrane protein CD36, which decreases during erythroid cell differentiation^16^, was first evaluated. CD36 levels decreased from day 6 of differentiation in WT and *HBB*^+/-^ cultures, but was retained in *HBB*^−/−^ cultures, correlating with arrested differentiation of *HBB*^−/−^ cells at the polychromatic stage (Supplementary Fig. 4), and providing effective resolution of transition from polychromatic to orthochromatic erythroblasts. Dual staining of CD36 with Annexin V then provided a robust and high throughput compatible assay for monitoring IE, as illustrated in Fig. 1i. To clarify, as differentiation proceeds WT and *HBB*^+/−^ cells show progressively decreasing levels of CD36 and consistently low levels of AnV, with the majority of cells by day 7 in quadrant 4, which delineates viable orthochromatic erythroblasts (CD36^low^AnV^−^; Fig. 1i and j). In contrast very few *HBB*^−/−^ cells proceed to quadrant 4, instead shifting from quadrant 1 to 2 (CD36^high^AnV^+^; Fig. 1i and k) as levels of apoptosis increase with cells reaching the polychromatic stage. The number of cells in Q2 (CD36^high^AnV^+^):Q4 (CD36^low^AnV^−^) provides an IE ratio and numerical value for the severity of IE (Fig. 1l). The second *HBB*^−/−^ clone showed similar profile and IE ratio in the assay (Supplementary Fig. 3D–F).

### Validating β^0^-thalassemia line as a drug and reagent screening platform

To test the potential of the *HBB*^−/−^ line as a screening platform for drugs and therapeutic approaches for reducing the disease severity of β-thalassaemia, we employed two established approaches that increase the level of γ-globin, and thus fetal hemoglobin (HbF) in erythroid cells. Upregulation of γ-globin was selected as our gauge, as it is the most promising approach for the treatment of β-thalassemia, and also sickle cell disease (SCD), compensating for loss of, or mutated, β-globin.

**Fig. 2:**
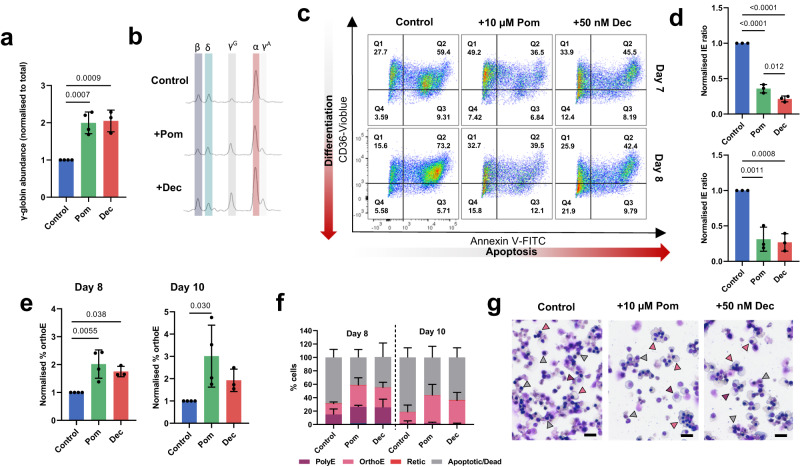
*HBB*^−/−^ cells respond to pomalidomide and decitabine treatment with increased γ-globin expression and reduced ineffective erythropoiesis. Differentiating *HBB*^−/−^ cells were treated with 10 µM pomalidomide (Pom), 50 nM decitabine (Dec) or vehicle only control (0.1% DMSO) and harvested for RP-HPLC analysis and IE analysis by flow cytometry. **a** Quantification of γ-globin levels (^G^γ-globin + ^A^γ-globin) at day 6 measured by RP-HPLC, normalized to total globin levels and shown relative to control samples. **b** Representative RP-HPLC traces. Peaks are identified for β-globin (β), δ-globin (δ), ^G^γ-globin (^G^γ), α-globin (α) and ^A^γ-globin (^A^γ). **c** Representative IE flow cytometry plots at day 7 and 8 of differentiation. **d** Quantification of IE ratio (%CD36^high^AnV^+^/CD36^low^AnV^−^). **e** Percentage of orthochromatic erythroblasts in Pom and Dec treated cultures normalized to Control cultures at day 8 and 10 of differentiation. **f** Morphology analysis of Pom and Dec treated HBB^−/−^ cells at day 8 and 10 of differentiation. **g** Representative cytospin images of Pom and Dec treated HBB^−/−^ cells at day 10 of differentiation. Arrowheads indicate the following cell types: purple, PolyE; pink, OrthoE; red, Retic; gray, dead/apoptotic. Scale bars 20 μm. Results show mean ± SD, *n* = 4 (control and Pom) *n* = 3 (Dec) independent experiments. Statistical significance was determined using one-way ANOVA with Tukey multiple comparison testing. Source data are provided as a Source Data file.

Pomalidomide and Decitabine have both been reported to increase γ-globin expression in human primary erythroid cells^17,18^. The *HBB*^−/−^ cells responded accordingly to both drugs, with significant increase in γ-globin levels (by RP-HPLC, Fig. 2a and b). To equate with effect on disease severity, the *HBB*^−/−^ cells were evaluated by our IE assay, which revealed a concurrent decrease in IE ratio (Fig. 2c and d), and increased number of cells terminally differentiating (Fig. 2e–g). Similar results were seen with hydroxyurea (HU) treatment, a drug approved for the treatment of non-transfusion dependent β-thalassemia and SCD, with increase in γ-globin levels and decrease in IE ratio paralleling increasing concentrations of HU (Supplementary Fig. 5).

We also used CRISPR-Cas9 gene editing to disrupt the +58 enhancer of the γ-globin repressor *BCL11A* in *HBB*^−/−^ cells, using a previously validated sgRNA^19^, an approach granted PRIME designation for TDT and SCD, with planned regulatory submission this year. A clonal population of +58 enhancer edited *HBB*^−/−^ cells containing a compound heterozygous deletion (Supplementary Fig. 6A) showed an ~25% increase in γ-globin levels (Supplementary Fig. 6B and C) in line with previous findings^20^, and a decrease in the severity of IE, as indicated by an ~50% decrease in the IE ratio at day 7 of differentiation and an increase in cell viability at day 8 and 10 of culture (Supplementary Fig. 6D–F). Improvement in erythroid differentiation was also observed, with a significant increase in the percentage of cells progressing to the orthochromatic stage at day 10 (Supplementary Fig. 6G–I).

These findings confirm that the *HBB*^−/−^ disease line responds effectively to both pharmacological and genome editing approaches to increase γ-globin expression, whilst also directly demonstrating that the treatments reduce IE and thus the severity of β^0^-thalassemia disease phenotype, collectively providing proof-of-principle for utilizing the line for screening applications.

### Generating β^0^-thalassemia cell lines with specific prevalent mutations

While the *HBB*^−/−^ lines do successfully recapitulate the expected phenotype of β^0^-thalassemia erythroid cells, the deletion mutation is not one of over 300 different mutations that cause reduced or absent β-globin production in β-thalassemia patients^21^. Therefore, we next selected two prevalent β^0^-thalassemia mutations to introduce into BEL-A, to more accurately replicate patient genotypes and corroborate data from *HBB*^−/−^ lines; CD41/42 -TTCT deletion which results in a frameshift and introduction of a premature stop codon and IVS-1-1 G → T substitution which prevents correct splicing of *HBB* mRNA^22^.

**Fig. 3:**
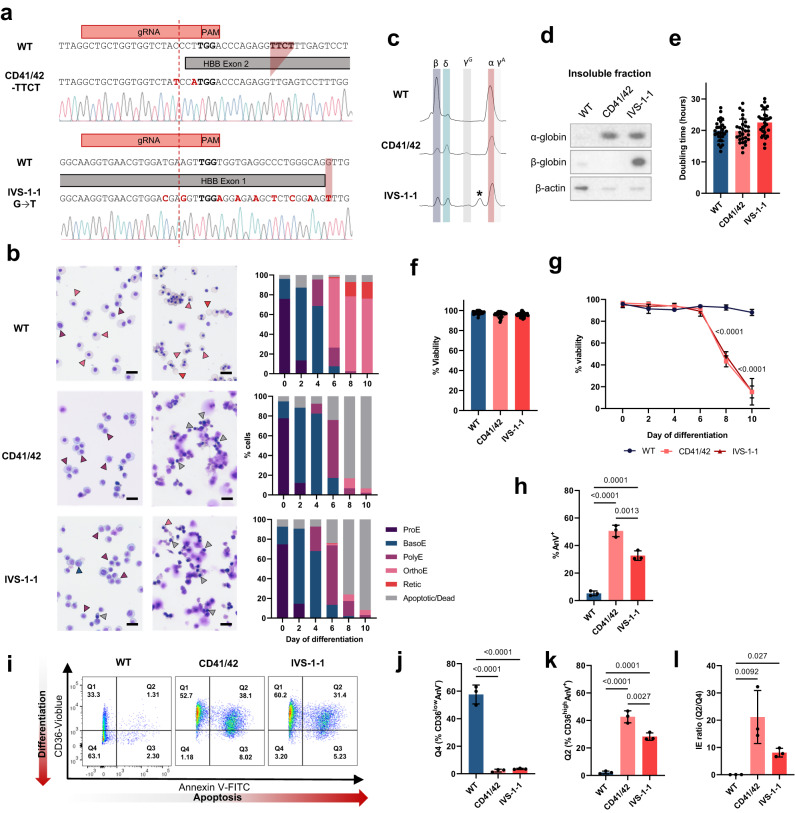
Generation and characterization β^0^-thalassemia cell lines with specific prevalent mutations. **a** Schematic of DNA sequence for CD41/42 -TTCT and IVS-1-1 G → T compared to WT BEL-A cells. **b** Representative cytospins of WT, CD41/42, and IVS-1-1 BEL-A with morphology quantification (ProE, proerythroblast; BasoE, basophilic erythroblast; PolyE, polychromatic erythroblast; OrthoE, orthochromatic erythroblast; Retic, reticulocyte). Arrowheads indicate the following cell types: blue, BasoE; purple, PolyE; pink, OrthoE; red, Retic; gray, dead/apoptotic. Scale bars 20 μm. **c** RP-HPLC traces for WT, CD41/42, and IVS-1-1 BEL-A at day 6 of differentiation. Peaks are identified for β-globin (β), δ-globin (δ), ^G^γ-globin (^G^γ), α-globin (α) and ^A^γ-globin (^A^γ). * Denotes splice β-globin variant (see Supplementary Fig. 7). **d** Representative western blot of aggregate proteins from WT, CD41/42, and IVS-1-1 BEL-A harvested at day 6 of differentiation, incubated with α-globin or β-globin antibody. β-actin was used as a protein loading control. Doubling time (**e**) and % viability (**f**) of expanding cell lines as determined by trypan blue exclusion assay, *n* = 32 independent time points (**g**) Percentage viability of WT, CD41/42, and IVS-1-1 BEL-A during differentiation by trypan blue exclusion assay. **h** Percentage of Annexin V positive (AnV^+^) cells in WT, CD41/42, and IVS-1-1 BEL-A at day 7 of differentiation as determined by flow cytometry. **i** Representative IE flow cytometry plots at day 7 of differentiation. Quantification Q4 CD36^low^AnV^-^ cells (**j**), Q2 CD36^high^AnV^+^ (**k**) and IE ratio (Q2/Q4) (**l**) at day 7 of differentiation. Results show mean ± SD, *n* = 3 independent experiments unless otherwise stated. Statistical significance was determined using one-way ANOVA with Tukey multiple comparison testing. Source data are provided as a  Source Data file.

Both homozygous mutations were successfully introduced into BEL-A using RNP-based CRISPR-Cas9 genome editing with a single-strand oligodeoxynucleotide (ssODN) donor template (Fig. 3a). In the case of IVS-1-1, due to the relatively large 24 base pair distance from the cleavage site of the closest possible *HBB*-specific sgRNA and the intended edit, the ssODN was recoded with silent mutations, which has been shown to enhance incorporation efficiency of distal edits^23^.

As for the WT line, both disease lines had a predominantly pro-erythroblast morphology with some basophilic erythroblasts (Fig. 3b).

Similar to the *HBB*^−/−^ line, β-globin protein was eliminated in both the CD41/42 and IVS-1-1 clonal lines (Fig. 3c), and α-globin reduced due to insoluble aggregation (Fig. 3c and d), with doubling times and viability during the expansion phase not significantly different (Fig. 3e and f).

As for the *HBB*^−/−^ lines, both the CD41/42 and IVS-1-1 lines demonstrated IE, with significantly reduced cell viability at the polychromatic cell stage (from day 6; Fig. 3b and g), due to significantly increased apoptosis compared to control cells (Fig. 3h), with very few cells surviving and differentiating to orthochromatic erythroblasts (Fig. 3g). Analysis using the flow cytometry IE assay showed similar profiles to the *HBB*^−/−^ line, with very few CD41/42 and IVS-1-1 cells shifted to quadrant 4 by day 7 (CD36^low^AnV^−^; Fig. 3i and j), instead having shifted to quadrant 2 (CD36^high^AnV^+^; Fig. 3i and k), due to levels of apoptosis increasing when cells reached the polychromatic stage, giving significantly increased IE ratios compared to WT cells (Fig. 3l).

The overall karyotypes of the three β-thalassemia lines (*HBB*^−/−^, CD41/42 and IVS-1-1) are similar to that of the original BEL-A line^16^, with trisomy of chromosome 6 and 19 and a small number of partial chromosome losses/abnormalities.

**Fig. 4:**
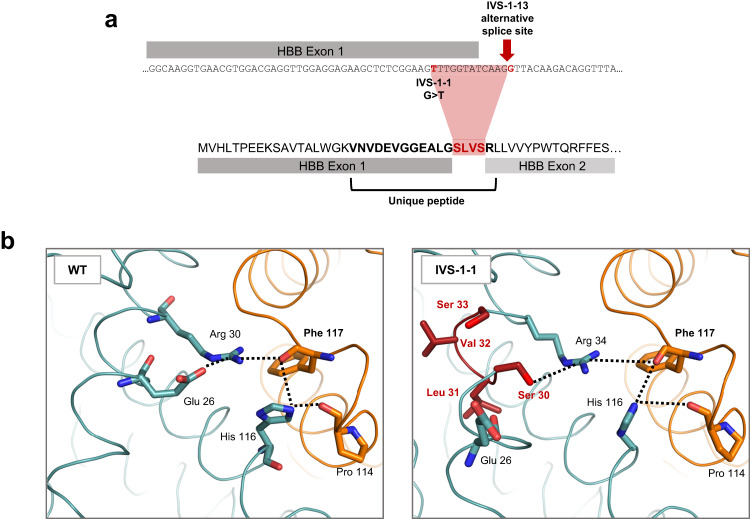
Identification of IVS-1-1 β-globin variant. **a** Schematic of the IVS-1-1 variant amino acid sequence resulting from use of the IVS-1-13 alternative splice site. The resulting peptide sequence contains a 4 amino acid insertion (red) from 12 base pairs of translated *HBB* intron. **b** Models of the α-/β-globin interface (α-globin in orange and β-globin in teal) showing increased distances between residues contributing to interactions with Phe 117 (dashed lines) on the α-globin chain in IVS1-1 compared to WT dimers. Inserted amino acids in the IVS-1-1 variant are shown in dark red.

Interestingly, an additional peak was observed in the IVS-1-1 line RP-HPLC traces (indicated by * in Fig. 3c). Mass spectrometry sequencing revealed this to be a splice variant of β-globin, created by splicing machinery using a previously identified IVS-1-13 alternative splice site^24^, resulting in a 4 amino acid insertion in β-globin between exon 1 and 2 (G29_R30insSLVS; Fig. 4a and Supplementary Fig. 7). Molecular modelling revealed disruption of interactions between the β-globin variant and α-globin, including with α-globin Phe 117, critical for α1β1 dimerization^25^, due to increased distances between the network of key residues (Fig. 4b). Of note, the variant β-globin was detected in the insoluble fraction of IVS-1-1 cells (Fig. 3d), further supporting the hypothesis that it is unable to form a stable hemoglobin tetramer with α-globin and thus precipitating, potentially contributing to the IE phenotype of β-thalassemia erythroid cells with the IVS-1-1 mutation.

### Culture of β-thalassemia patient cells further validates β-thalassemia cell lines

Due to the limited availability of suitable patient material for in vitro studies, there are few reports analyzing cultures of β-thalassemia patient erythroid cells^2,3^. To provide further analysis, and to validate our model β-thalassemia lines under the same conditions, peripheral blood mononuclear cells (PBMCs) from two β-thalassemia patients (IVS-1-110 G → A [β^+^] and IVS-1-1 G → T [β^0^]) were isolated and differentiated using our erythroid culture system. As seen with the β-thalassemia lines, and as reported previously for in vitro cultured β-thalassemia (β^0^) erythroid cells^2^, there was drastically increased cell death and onset of apoptosis correlating with differentiation to polychromatic erythroblasts. This is exemplified using the IE assay, with increased IE ratio clearly apparent from day 11 of differentiation onwards (Supplementary Fig. 8A–D). The IE assay distinguished degree of severity between the β^0^ (IVS-1-1) and β^+^ (IVS 1-110) patient erythroid cells, with the IE ratio corresponding with genotype (Supplementary Fig. 8B). In addition, α-globin was found at high abundance in the insoluble fraction of whole cell lysates from differentiated cells (Supplementary Fig. 8E), due to aggregation in the absence of sufficient β-globin. The mutant β-globin identified in the IVS-1-1 cell line, was also detected in the IVS-1-1 (β^0^) patient erythroid cells (Supplementary Fig. 8E) and established as the same splice variant by mass spectrometry, confirming that this mutated β-globin is indeed produced in patients.

### Quantitative proteomic comparison of WT and *HBB*^−/−^ BEL-A

To obtain a global assessment of differences between WT and *HBB*^−/−^ erythroid cell proteome, verify alterations in proteins previously identified, and reveal novel proteins and processes for potential therapeutic targeting, TMT LC-MS/MS comparative proteomic analysis was performed. For this, cells were evaluated during early differentiation (basophilic erythroblasts), prior to manifestation of IE phenotype, and in mid-differentiation (polychromatic erythroblasts), when the α-/β-globin chain imbalance approaches maximal levels and apoptosis is induced. Due to differences in kinetics of the WT and *HBB*^−/−^ cultures, basophilic and polychromatic erythroblasts were isolated by FACS, using inverse levels of GPA and CD36 (representative cytospin images of these populations are shown in Supplementary Fig. 9).

**Fig. 5:**
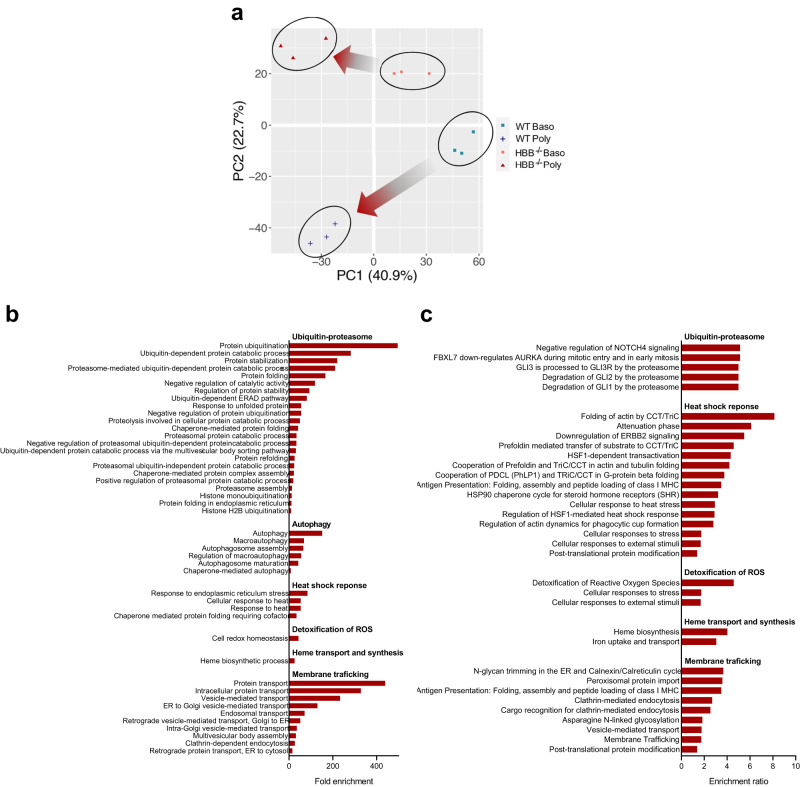
Comparative proteomic analysis of *HBB*^−/−^ and WT BEL-A. **a** Principal component analysis (PCA) plot of multiplex TMT-based comparative proteomics of FACS-isolated basophilic and polychromatic erythroblasts from *HBB*^−/−^ and WT BEL-A. **b** and **c** Overrepresented pathways in *HBB*^−/−^ vs WT BEL-A polychromatic cells from TMT-based comparative proteomic data. Pathways shown were significantly enriched by FDR ( < 0.05) from (**b**) DAVID (**c**) Webgestalt proteomics pathway analysis tools. Source data are provided as a Source Data file.

Of the 3364 unique proteins quantified, 1048 (31%) were significantly different in *HBB*^−/−^ compared to WT basophilic erythroblasts, and 1001 (30%) in *HBB*^−/−^ compared to WT polychromatic erythroblasts (Supplementary Data 1). Despite the similar proportion of significantly different proteins in both cell types, principal component analysis (PCA; Fig. 5a) illustrates the increased magnitude of proteome changes between WT and *HBB*^−/−^ polychromatic erythroblasts compared to basophilic erythroblasts, reflecting the more severe phenotype of the former.

Focussing on the polychromatic erythroblasts where changes in proteome were more pronounced, we interrogated the set of proteins significantly up-regulated and significantly down-regulated in *HBB*^−/−^ cells, also using Gene Ontology and Pathway Enrichment Analysis toolkits WebGestalt^26^ and DAVID^27^ to identify overrepresented pathways. No down-regulated pathways reached significance, but multiple significantly upregulated pathways were identified, as detailed below.

**Fig. 6:**
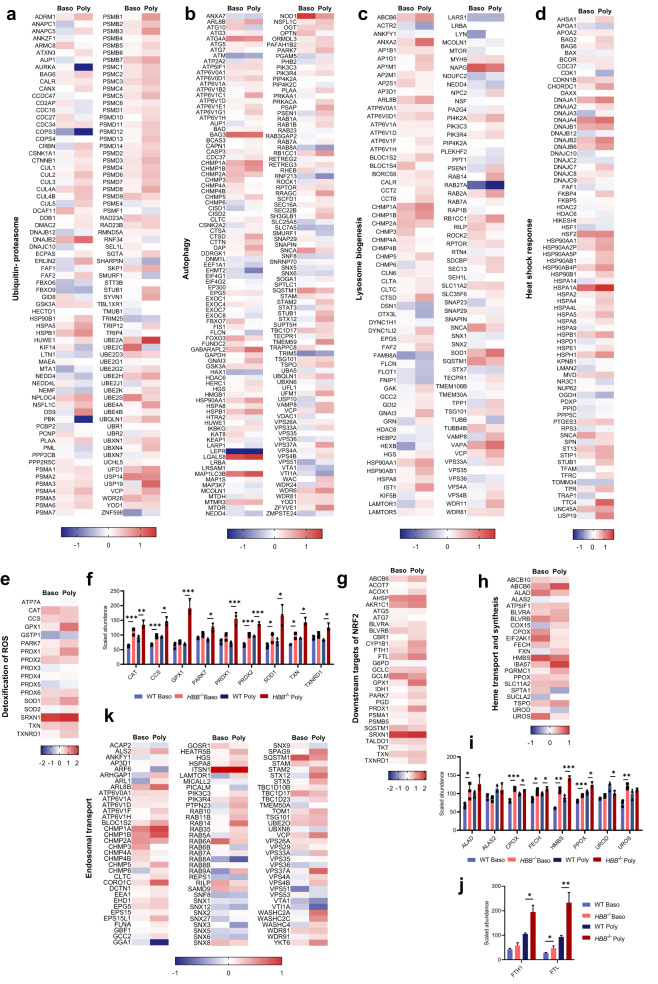
Analysis of alterations in abundance of proteins from significantly enriched pathways of *HBB*^−/−^ vs WT BEL-A. Heatmaps show log_2_ fold-changes (FC) of all WT vs *HBB*^−/−^ normalized protein abundances in both basophilic (Baso) and polychromatic (Poly) erythroblasts from significantly upregulated pathways identified by pathway analysis, using the following search terms on http://geneontology.org/: (**a**) ubiquitin-proteasome, (**b**) autophagy, (**c**) lysosome biogenesis, (**d**) heat shock response, (**e**) detoxification of reactive oxygen species (ROS), (**h**) heme transport and synthesis, and (**k**) endosomal transport. **g** Heat map of all downstream target proteins of NRF2 identified in the dataset. Bar charts show abundance values normalized to total protein and scaled relative to 100 shown as mean ± SD, *n* = 3 independent experiments, for antioxidant enzymes (**f**), Heme processing enzymes (**i**), and ferritin heavy and light chain (**j**). *P < 0.05, **P < 0.01, ***P < 0.001. *P* values represent results from two-tailed heteroscedastic *t*-tests performed on log_2_ normalized data. Source data are provided as a Source Data file.

β-thalassemia has been described as a protein-aggregation disorder^28^ with increased activity of the ubiquitin-proteasome pathway and autophagy^29–31^ as cells attempt to clear the α-globin aggregates. Here we show that the ubiquitin-proteasome and autophagy pathways are significantly upregulated in *HBB*^−/−^ polychromatic erythroblasts (Fig. 5b and c), with the majority of proteins identified in our dataset involved in these pathways increased in level (Fig. 6a and b). In addition, the abundance of the majority of proteins in the lysosome biogenesis pathway, associated with autophagy, was increased (Fig. 6c). We also found enrichment of pathways associated with heat shock response (Fig. 5b), with a striking increase in the majority of identified proteins including many chaperones (Fig. 6d). Changes in abundance of proteins within these pathways were also detected in basophilic erythroblasts, although in general were less apparent than in polychromatic erythroblasts (Fig. 6), in line with increasing α-globin levels.

Of note, the most significantly over-expressed chaperone protein in both *HBB*^−/−^ polychromatic and basophilic erythroblasts was HSP70 (HSPA1A; Supplementary Fig. 10A). HSP70 has previously been reported to interact with aggregated α-globin in β-thalassemia erythroblasts sequestering it to the cytosol, thus preventing its translocation to the nucleus to protect GATA1 from degradation^3^. Consistent with this finding, GATA1 levels were significantly decreased in *HBB*^−/−^ basophilic and polychromatic erythroblasts (Supplementary Fig. 10A) and also in the nuclear fraction of these cells (Supplementary Fig. 10B and C). In addition, HSP70 was found to colocalise with α-globin in the insoluble fraction of *HBB*^−/−^ cell lysate (Supplementary Fig. 10D) and in the insoluble fraction of β-thalassemia patient primary erythroid cell lysates (Supplementary Fig. 8E).

Few pathways involved in oxidative stress have been studied in detail in β-thalassemia erythroid cells^32,33^ although auto oxidation of α-globin aggregates is hypothesized to lead to increased ROS^34^. Consistent with this, pathways associated with detoxification of ROS were significantly overrepresented (Fig. 5b and c), with the majority of proteins involved in detoxification of ROS found at increased levels (Fig. 6e), including eight antioxidant enzymes significantly increased in polychromatic erythroblasts, with four also significantly increased in basophilic erythroblasts, prior to onset of apoptosis (Fig. 6f). In addition, target genes of NRF2^35–37^, an activator of the antioxidant response programme^38^ previously shown to be upregulated in β^IVS-2-654^ thalassaemic mice^37^, were increased (Fig. 6g).

Interestingly, pathways involved in heme transport and synthesis were overrepresented in our dataset (Fig. 5b and c), with proteins involved in heme biosynthesis found at overall higher levels in both *HBB*^*−*/−^ basophilic and polychromatic erythroblasts (Fig. 6h). This included seven of the eight core heme synthesis enzymes, with just one in basophilic and two in polychromatic erythroblasts failing to reach significance (Fig. 6i). Levels of both ferritin light chain and ferritin heavy chain were also significantly increased (Fig. 6j). In relation, pathways associated with endosomal transport and membrane trafficking were significantly enriched in polychromatic erythroblasts (Fig. 5b and c) and the majority of proteins involved in endosomal transport found at increased levels (Fig. 6k), indicating that more general transport mechanisms may be upregulated in order to elevate heme transport capacity. To further investigate the increased heme biosynthesis observed in β-thalassemia erythroblasts, WT and *HBB*^*−*/−^ cells were treated with 50 µM succinylacetone during differentiation to inhibit the second step of heme synthesis. Succinylacetone significantly reduced the viability of WT cells, but conversely did not affect viability of *HBB*^−/−^ cells. Instead there was a small but significant decrease in CD36 levels indicating increased maturation of the *HBB*^*−*/−^ cells. (Supplementary Fig. 11). These data indicate that heme biosynthesis is upregulated above functionally required levels in β-thalassemia erythroid cells, and that normalizing levels may have a therapeutic effect. Overall, the findings suggest that increased heme biosynthesis in β-thalassemia erythroblasts, likely in response to decreased hemoglobin levels, could exacerbate IE as well as erythroid intrinsic iron overload, contributing to the disease phenotype.

## Discussion

In this study we created, characterized, and validated human disease cellular model systems for β-thalassemia, which recapitulate the phenotype of patient erythroid cells. The lines provide a sustainable and consistent supply of disease cells as unique research tools for studying the underlying molecular defects, for identifying new therapeutic targets, and as screening platforms for drug and therapeutic reagents. For the latter we also developed a high throughput compatible fluorometric-based assay for evaluation of severity of IE, and thus of disease phenotype. We then utilized the assay to demonstrate our β-thalassemia lines respond appropriately to drug and gene therapy approaches previously shown to increase γ-globin, with resultant improvement to IE, providing validation for such applications.

Proteomic studies have previously been utilized to identify potential biomarkers of β-thalassemia disease severity, analyzing serum^39^, plasma^40–43^, or peripheral blood microvesicles^44,45^, for improved clinical management. Such analyses, although important, address downstream sequelae of the root cause of disease pathophysiology, IE of the erythroid cells. Our β-thalassemia disease lines overcome the hurdle of obtaining adequate numbers of bone marrow erythroid cells or haematopoietic stem cells from patients, enabling in depth and accurate analysis of molecular changes underlying the defective differentiation. In addition, an issue with comparing control and β-thalassemia erythroid cells arises from differences in kinetics of the cultures^2^ (Figs. 1b and 3b), whereby differences due to stage of differentiation can confound identification of those due to disease phenotype. However, as we show, our lines enable sufficient size cultures to isolate specific, stage-matched cells.

Using TMT-based comparative proteomics we reveal the changes in biological pathways and processes in β-thalassemia erythroid cells, including upregulated pathways associated with, and increased levels of, antioxidant response enzymes. In addition, we identified increased heat shock proteins and chaperones, and increased levels of many proteins of the ubiquitin-proteasome and autophagy pathways along with proteins of associated lysosome biogenesis, together with respective upregulated pathways. The changes in proteome are manifest in earlier erythroid cells, but with the magnitude of change increased by the polychromatic erythroid cell stage, in line with increased levels of α-globin. However, this plethora of cellular responses are unable to adequately remove the aggregated α-globin or resolve the downstream sequelae, resulting in manifestation of the disease phenotype.

Previously, only few proteins associated with some of these processes have been reported upregulated in β-thalassemia erythroid cells^29,31,46^, although increased abundance of transcripts for most proteosome subunits has been reported in the β-globin^Th3/+^ and β-globin^Th3/Th3^ mouse^30^, in line with increased levels of these proteins in human cells in the present study. In contrast, our data reveal extensive up regulation of proteins involved in wide range of biological response pathways, in a biologically relevant human system.

We also reveal upregulated pathways and increased levels of proteins for heme biosynthesis, including all eight core heme synthesis enzymes, along with associated upregulated endosome biogenesis and trafficking pathways. These findings support the hypothesis that erythroid-intrinsic iron overload may be contributing to oxidative stress in β-thalassemia, as heme molecules bind insoluble α-globin chains exacerbating the toxicity of these aggregates^47^. Heme also inhibits HRI, increasing globin synthesis, which will cause further aggregate formation^48^. Succinylacetone is an inhibitor of the second step of heme synthesis and has shown promise in alleviating disease phenotype in Diamond Blackfan Anemia (DBA) erythroid cells in vitro^49^. Our data show that succinylacetone treatment also has a positive, albeit small, effect on β-thalassemia erythroid cells. The detrimental effect of the drug on viability of WT, but not β-thalassemia erythroid cells, supports the pathological upregulation of heme biosynthesis observed in the *HBB*^−/−^ erythroid proteomic data. More targeted strategies to reduce heme synthesis may provide improved effects and thus potential novel avenues for therapeutic intervention in β-thalassemia. For example, catalytic activity of erythroid specific ALAS2, the first enzyme in the heme synthesis pathway, can be modulated by small molecules binding a hotspot around its autoinhibitory Ct-extension^50^.

Amongst the 10 most aberrantly increased proteins in β-thalassemia erythroid cells (*HBB*^−/−^; Supplementary Table 1) those with a possible causative role in disease phenotype, rather than resultant change, and thus potential application as novel therapeutic targets for follow up, include: OTUD5 a deubiquitinase, increased levels of which could exacerbate production of α-globin aggregates directly and thus ineffective erythropoiesis, and also via mTORC1 pathway, OTUD5 being a positive regulator of mTORC, with inhibition of mTORC1 increasing autophagy and reducing α-globin aggregation and disease phenotype in a mouse model of β-thalassemia;^31^ Cathepsin B (CTSB), a negative regulator of autophagy, also over expressed in Sickle Cell Disease erythroid cells where it is being investigated as a therapeutic target;^51^ Exportin 7 (XPO7) a broad-spectrum exportin and a nuclear import receptor implicated in erythroid differentiation, with increased levels potentially perturbing critical intracellular processes^52^. Proteins that are reduced in level are more challenging to target therapeutically, but of interest ZNF648 was ~6-fold lower (*p* < 0.0001) in β-thalassemia polychromatic erythroid cells, with reduced levels of ZNF648 in wild type erythroid cells shown to impede erythroid cell survival and differentiation^53^.

As aberrant activation/inactivation of proteins can also perturb intracellular processes we also compared the phosphorylation status of proteins in the proteomic datasets. Proteins with greatest alteration in phosphorylation status (twofold or more in *HBB*^−/−^ compared to WT polychromatic erythroid cells) are shown in Supplementary Table 2, along with peptide and residues phosphorylated. Although the function of many of these proteins in erythropoiesis is presently unknown, such data has potential to provide information on changes that lead to ineffective erythropoiesis, follow up analyses also distinguishing causative from consequential effects on phosphorylation status. For example, the GTPase RAB8A had significantly increased phosphorylation at S111 in β-thalassemia erythroid cells, a modification which inactivates this GTPase^54^, disrupting associated signalling cascades; OSBP2 has been implicated as potential driver of erythroid cell differentiation^55^, with phosphorylation modulating its function; Slc29a1 promotes erythroblast survival and erythroid differentiation^56^, and is known to be phosphorylated at multiple sites by both PKA and PKC^57^, however, how phosphorylation regulates the function of this adenosine transporter is not yet known. Proteins with significantly reduced levels of phosphorylation were found to be predominantly associated with cellular stress and apoptosis pathways, likely consequential changes, as well as with the cell cycle.

CRISPR editing to KO the *HBB* gene in the HUDEP-2 erythroid cell line has been shown to result in a significant increase in γ-globin or HbF due to a stress erythropoiesis response^33,58^. However, increased γ-globin expression is not an inherent feature of β-thalassemia. Indeed, β-thalassemia patients who co-inherit other genetic variants that cause increased γ-globin expression and HbF have reduced or ameliorated disease severity (reviewed by Vinjamur et al. ^59^), with methods to increase HbF the most promising therapeutic approaches for the disease. A β-thalassemia phenotype with α-globin aggregates and IE was not reported for these HUDEP-2 KO lines. In contrast, Zhang et al. ^60^ did not get increased γ-globin following differentiation, when instead used prime editing to introduce β-thalassemia mutations into the *HBB* gene of HUDEP-2 cells, with most lines having lower expression of γ-globin than the unedited control cells. However, introduction of even silent mutations, that do not result in a β-thalassemia disease phenotype, into the *HBB* gene of HUDEP-2 by prime editing resulted in significant apoptosis and cell death of the resultant lines when differentiated, as much or even more so than lines with mutations that cause β^0^ thalassemia introduced. The cell death therefore appears to be associated with the prime editing process itself, with the lines not therefore representing a β-thalassemia phenotype.

Intriguingly, we found a novel mutant β-globin splice variant arising from the IVS-1-1 G → T mutation, predicted to reduce the stability of dimer and tetramer and thus still resulting in a β^0^-thalassemia phenotype. A transcript for this variant has previously been reported from overexpressing IVS-1-1 G → A *HBB* coding region construct in HeLa cells^24^. That the variant does not have a beneficial role correlates with the lack of evidence for a milder clinical phenotype in IVS-1-1 G → T patients compared to those with frameshift mutations^61^. In addition, there was no evidence of reduced insoluble α-globin in our IVS-1-1 compared to CD41/42 line, further supporting the hypothesis that the IVS-1-1 β-globin variant cannot form stable interactions with α-globin. Instead, as the variant β-globin precipitates it could exacerbate the disease phenotype. As there are many splicing mutations that cause β-thalassemia, it will be interesting to determine if other in-frame mutant β-globin splice variants are produced and if so, how they impact disease phenotype.

Finally, the findings presented here also provide proof of principle for CRISPR-Cas9 genome editing of BEL-A to create sustainable model cellular systems of other red blood cell disorders, expanding the toolkit available to erythropoiesis researchers.

## Methods

### Cell culture

#### Primary cells

Peripheral blood mononuclear cells (PBMCs) were isolated from LRS cones for control cells and whole blood for β-thalassemia cells. LRS cones were obtained with informed consent from all donors and used in accordance with the Declaration of Helsinki and approved by the National Health Service National Research Ethics Committee (reference number 08/H0102/26) and the Bristol Research Ethics Committee (reference 12/SW/0199). Whole blood from β-thalassemia patients was obtained under ethics board North of Scotland REC (18/NS/0005). PBMCs were thawed into EDM supplemented with 1 ng.ml^−1^ IL‐3 and 10 ng.ml^−1^ SCF and a lineage depletion was performed on day 5 according to manufacturer’s instructions (Miltenyi Biotec; 130-092-211). On day 9, cells were transferred to EDM supplemented with 10 ng.ml^−1^ SCF only, and on day 13 cells were transferred to EDM supplemented with holo-transferrin to a final concentration of 500 µg.ml^−1^.

#### BEL-A

BEL-A cells were cultured as previously described^62^. In brief, for expansion phase cells were cultured in StemSpan™ SFEM [Stemcell Technologies] containing 50 ng.ml^−1^ SCF, 3 U.ml^−1^ EPO, 1 μM dexamethasone and 1 µg.ml^−1^ doxycycline. To induce differentiation, expanding cells were transferred to erythroid differentiation medium (Iscove’s medium with stable glutamine [Merck] containing 3% (v/v) AB serum [Merck], 2% (v/v) fetal bovine serum [Hyclone], 10 µg.ml^−1^ Insulin [Merck], Heparin 3U.ml^−1^ [Merck], 200 µg.ml^−1^ holo-transferrin [Sanquin, NED] and 3U.ml^−1^ EPO [Roche]—EDM) supplemented with 1 ng.ml^−1^ IL‐3 and 10 ng.ml^−1^ SCF (both R&D Systems) and 1 µg.ml^−1^ doxycycline for 4 days, and for a further 4 days without doxycycline. Cells were then transferred to EDM supplemented with holo-transferrin to a final concentration of 500 µg.ml^−1^. For hydroxyurea treatment, hydroxyurea (H8627; Sigma) was added to cells at 50 μM every 2 days from 4 days prior to the start of differentiation until harvesting of cells for analysis. Pomalidomide (10 μM; P0018; Sigma-Aldrich) was added every 2 days and Decitabine (50 nM; A3656; Sigma-Aldrich) was added to cells daily during differentiation.

### CRISPR-Cas9 genome editing of BEL-A

#### Plasmid-based editing

To generate *HBB*^−/−^ and *HBB*^+/−^ lines, Guide RNA oligos (GTAACGGCAGACTTCTCCTC) were ligated into the pSpCas9(BB)-2A-GFP (px458) vector (48138; Addgene). BEL-A cells (1.0 × 10^6^) were resuspended in CD34+ nucleofection kit buffer (Lonza Biosciences) containing 2.5 µg px458 (with gRNA) and 2.5 µM ssODN donor template, and electroporated using program U-008 of AMAXA Nucleofector™ 2b. After 48 h, dead and dying cells were removed using a dead cell removal kit (130-090-101; Miltenyi). After a further 24 h, single-cell cloning of GFP + /DRAQ5- cells into 96-well plates was performed using a BDInflux Cell Sorter.

#### Ribonucleoprotein (RNP)-based editing

A sub-cloned population of BEL-A cells (1.0 × 10^5^) were resuspended in CD34+ nucleofection kit buffer (Lonza Biosciences) containing 18 pmol Cas9, 45 pmol gRNA. To generate the *HBB*^−/−^ *HBD*^−/−^ line, three gRNAs previously validated by Boontanrart et al.^58^. were used simultaneously, two of which were specific to *HBB* (e87 (g10) CUUGCCCCACAGGGCAGUAA; e383 GCUCAUGGCAAGAAAGUGCU), and one of which targeted both *HBB* and *HBD* (e297 UGGUCUACCCUUGGACCCAG). For CD41/42 -TCTT IVS1-1 G → T *HBB* edits, gRNAs ((CD41/42 *HBB*—GGCUGCUGGUGGUCUACCCU; IVS1-1 *HBB*—AAGGUGAACGUGGAUGAAGU, Synthego) were used along with 100 pmol ssODN template containing the mutation of interest. ssODN templates were antisense for both the CD41/42 and IVS1-1 edits with homology arms 36 nucleotides away from the edit and 91 nucleotides toward the edit, in accordance with the design principles described previously^23^. A recoded template was chosen for IVS1-1 G → T, where silent mutations are incorporated into the ssODN between the site of the DSB and the intended edit to increase editing efficiency^23^, due to the relatively large distance from the edit to DSB.

*BCL11A* + 58 enhancer editing, was performed as above with *HBB*^−/−^ BEL-A as a founder population using a previously validated gRNA targeting the *BCL11A* + 58 enhancer (CUAACAGUUGCUUUUAUCAC)^2,13^.

### Clone screening

Genomic DNA was isolated and the gene region of interest was amplified using GoTaq DNA polymerase (Promega) (*HBB* Fw: 5′- TGGTATGGGGCCAAGAGATA-3′, *HBB* Rv: 5′-GAGCCAGGCCATCACTAAAG-3′; *BCL11A* + *58* Fw: GGCAGCTAGACAGGACTTGG, *BCL11A* + *58* Rv: GGAGGCAAGTCAGTTGGGAA). PCR products were cleaned up (QIAquick PCR purification Kit, Qiagen) and sequenced by EuroFins Scientific. Sequencing data were analyzed by TIDE^63^ and TIDER^64^ web tools (https://tide.nki.nl/).

### Cytospin preparation, imaging, and morphology analysis

Aliquots of 2–8 × 10^4^ cells in 200 µl of appropriate cell culture medium were used to prepare cytospin preparations on coated slides, by centrifugation at 400 *g* for 5 min with a Thermo Scientific Shandon 4 Cytospin. The slides were stained in Leishman’s Eosin-Methylene blue solution (VWR). The stained slides were imaged using an Olympus CX43 microscope mounted with Olympus SC50 camera. Morphology analyses were determined by counting at least 200 cells per slide.

### Flow cytometry

#### Ineffective erythropoiesis (IE) assay

Aliquots of 2–3 × 10^5^ cells were incubated with CD36-Vioblue conjugated antibody (Clone AC106, 130-095-482; Miltenyi; dilution 1:11) in PBS containing 1% (w/v) BSA (Park Scientific Ltd) and 2 mg.ml^−1^ glucose (PBS-AG), followed by incubation with Annexin V-FITC conjugated antibody (130-093-060; Miltenyi; dilution 1:11) in Annexin V binding buffer (10 mM Hepes, pH7.4; 140 mM NaCl; 2.5 mM CaCl_2_). Cells were analyzed on a BD LSR Fortessa or BD LSR II flow cytometer and data analyzed using FlowJo v10.6.1 (FlowJo LLC). Gating strategy is depicted in Supplementary Fig. 12.

### RP-HPLC

RP-HPLC was performed as described by Loucari et al.^65^ with the exception of the injection size (600,000 cells in 30 µl dH_2_O) and the LC column used (Jupiter 5 µm C18 300 A, size 250 × 4.4 mm, protected with a Security Guard analytical guard system [KJ0-4282, Phenomenex]). Purified HbA2 and HbA0 controls (H0266 and H0267; Sigma Aldrich) were reconstituted in dH_2_O at 200 ng/µl, with 30 µl used per run.

### SDS-PAGE and western blot

Proteins (either from whole cell lysates or insoluble fraction) were resolved by SDS-PAGE and transferred to PVDF membrane (Merck) by western blotting. The insoluble fraction was obtained by centrifugation (17,000 *g*) of whole cell lysate homogenized in RIPA buffer, before direct resuspension in SDS-PAGE sample buffer, with the equivalent of 250,000 cells loaded per lane. To obtain nuclear lysates, 1 × 10^7^ cells were resuspended with 800 µl of 4 °C HLBN buffer (10 mM Tris-HCl [pH 7.5], 10 mM NaCl, 2.5 mM MgCl_2_, 0.5% (v/v) nonyl phenoxypolyethoxylethanol-40 [NP-40]) and incubated for 5 min on ice. 200 µl HLBN buffer + 10% sucrose was then pipetted to under-lay the HLBN suspension. Following centrifugation at 400 *g* for 5 min, the supernatant was removed, and the remaining nuclear pellet was lysed in RIPA buffer. For each lane, 3 µg of whole cell lysate and 20 µg nuclear lysate was loaded. Membranes were blocked with 5% (w/v) milk powder before incubation in primary and HRP-secondary antibodies. Primary antibodies used were: Monoclonal mouse anti-hemoglobin α (Clone D-4, Cat. No. sc-514378, Lot No. I0916, Santa Cruz; dilution 1:3000), monoclonal mouse anti-hemoglobin β (Clone 37-8, Cat. No. sc-21757, Lot No. G2418, Santa Cruz; dilution 1:3000), monoclonal mouse anti-hemoglobin γ-globin (Clone 51-7, Cat. No. sc-21756, Lot No. G0318, Santa Cruz; dilution 1:1500), monoclonal rat anti-GATA1 (Clone N1, Cat. No. sc-266, Lot No. E1713, Santa Cruz; dilution 1:1500), polyclonal rabbit anti-Lamin A/C (Clone H-110, Cat. No. sc-20681, Lot No. B0111, Santa Cruz; dilution 1:1000), monoclonal mouse anti-β-actin (AC-15, Cat. No. A1978, Batch No. 0000086303, Merck; dilution 1:3000), monoclonal mouse anti-Ctip1/BCL-11A (Clone 14B5, Cat. No. ab19487, Lot No. GR3383959-1, Abcam; dilution 1:2000), mouse anti-CD233/Band3 (Clone BRIC 170, IBGRL; dilution 1:500), polyclonal rabbit anti-HSP70 (Cat. No. Cat. No. ADI-SPA-812-D, Lot No. 09011646, Enzo Life Sciences; dilution 1:1000). Secondary antibodies used were: Polyclonal rabbit anti-mouse immunoglobulins-HRP conjugated (Cat. No. P0260, Lot No. 41424309, DAKO; dilution 1:3000), polyclonal swine anti-rabbit immunoglobulins-HRP conjugated (Cat. No. P0399, Lot No. 41270083, DAKO; dilution 1:3000), polyclonal goat anti-rat immunoglobulins-HRP conjugated (Cat. No. 112-035-003, Jackson ImmunoResearch; dilution 1:10000). Bands were visualized using enhanced chemiluminescence (G.E. Healthcare) with a G:BOX Chemiluminescence imager (Syngene).

### Cell stage matching for comparative proteomics

Mouse anti-Glycophorin A (GPA; Clone BRIC 256; IBGRL; dilution 1:1), vs CD36 cell surface marker analysis by flow cytometry was used to isolate stage-matched basophilic and polychromatic erythroblasts for WT and *HBB*^−/−^ BEL-A cells. Cells were obtained from 3 independent cultures.

### TMT labelling, mass spectrometry and data analysis

Aliquots of 100 µg of protein digested with trypsin (2.5 μg trypsin; 37 °C, overnight) and labelled with Tandem Mass Tag (TMT) Pro tag reagents according to the manufacturer’s protocol (Thermo Fisher Scientific, Loughborough, LE11 5RG, UK), and the labelled samples pooled. For the Total proteome analysis, an aliquot of 50ug of the pooled sample was desalted using a SepPak cartridges according to the manufacturer’s instructions (Waters, Milford, MA, USA). Eluate from the SepPak cartridge was evaporated to dryness and resuspended in buffer A (20 mM ammonium hydroxide, pH 10) prior to fractionation by high pH reversed‐phase chromatography using an Ultimate 3000 liquid chromatography system (Thermo Fisher Scientific). In brief, the sample was loaded onto an XBridge BEH C18 Column (130 Å, 3.5 μm, 2.1 × 150 mm, Waters, UK) in buffer A and peptides eluted with an increasing gradient of buffer B (20 mM ammonium hydroxide in acetonitrile, pH 10) from 0 to 95% over 60 min. The resulting fractions were evaporated to dryness and resuspended in 1% formic acid prior to analysis by nano‐LC MSMS using an Orbitrap Fusion Tribrid mass spectrometer (Thermo Scientific).

For the Phospho-proteome analysis, the remainder of the TMT-labelled pooled sample was also desalted using a SepPak cartridge (Waters, Milford, Massachusetts, USA). Eluate from the SepPak cartridge was evaporated to dryness and subjected to TiO2-based phosphopeptide enrichment according to the manufacturer’s instructions (Thermo Fisher Scientific). The flow-through and washes from the TiO2-based enrichment were then subjected to FeNTA-based phosphopeptide enrichment according to the manufacturer’s instructions (Thermo Fisher Scientific). The phospho-enriched samples were evaporated to dryness and resuspended in 1% formic acid prior to analysis by nano-LC MSMS using an Orbitrap Fusion Tribrid mass spectrometer (Thermo Scientific).

High pH RP fractions (Total proteome analysis) or the phospho-enriched fractions (Phospho-proteome analysis) were further fractionated using an Ultimate 3000 nano‐LC system in line with an Orbitrap Fusion Tribrid mass spectrometer (Thermo Scientific). In brief, peptides in 1% (vol/vol) formic acid were injected onto an Acclaim PepMap C18 nano‐trap column (Thermo Scientific). After washing with 0.5% (vol/vol) acetonitrile 0.1% (vol/vol) formic acid, peptides were resolved on a 250 mm × 75 μm Acclaim PepMap C18 reverse‐phase analytical column (Thermo Scientific) over a 150‐min organic gradient, using seven gradient segments (1–6% solvent B over 1 min, 6–15% B over 58 min, 15–32% B over 58 min, 32–40% B over 5 min, 40–90% B over 1 min, held at 90% B for 6 min and then reduced to 1% B over 1 min) with a flow rate of 300 nl/min. Solvent A was 0.1% formic acid, and Solvent B was aqueous 80% acetonitrile in 0.1% formic acid. Peptides were ionized by nano‐electrospray ionization at 2.0 kV using a stainless‐steel emitter with an internal diameter of 30 μm (Thermo Scientific) and a capillary temperature of 275 °C.

All spectra were acquired using an Orbitrap Fusion Tribrid mass spectrometer controlled by Xcalibur 2.1 software (Thermo Scientific) and operated in data‐dependent acquisition mode using an SPS‐MS3 workflow. FTMS1 spectra were collected at a resolution of 120,000, with an automatic gain control (AGC) target of 200,000 and a max injection time of 50 ms. Precursors were filtered with an intensity threshold of 5000, according to charge state (to include charge states 2–7) and with monoisotopic precursor selection. Previously interrogated precursors were excluded using a dynamic window (60 s ± 10 ppm). The MS2 precursors were isolated with a quadrupole mass filter set to a width of 1.2 m/z. ITMS2 spectra were collected with an AGC target of 10,000, max injection time of 70 ms and CID collision energy of 35%.

For FTMS3 analysis, the Orbitrap was operated at 50,000 resolution with an AGC target of 50,000 and a max injection time of 105 ms. Precursors were fragmented by high energy collision dissociation (HCD) at a normalized collision energy of 60% to ensure maximal TMT reporter ion yield. Synchronous precursor selection (SPS) was enabled to include up to ten MS2 fragment ions in the FTMS3 scan.

The raw data files were processed and quantified using Proteome Discoverer software v2.4 (Thermo Scientific) and searched against the UniProt Human database (downloaded January 2022: 178,486 entries) using the SEQUEST algorithm. Peptide precursor mass tolerance was set at 10 ppm, and MS/MS tolerance was set at 0.6 Da. Search criteria included oxidation of methionine ( + 15.995 Da), acetylation of the protein N-terminus ( + 42.011 Da) and methionine loss plus acetylation of the protein N-terminus (−89.03 Da) as variable modifications and carbamidomethylation of cysteine ( + 57.0214) and the addition of the TMTpro mass tag ( + 304.207) to peptide N-termini and lysine as fixed modifications. For the Phospho-proteome analysis, phosphorylation of serine, threonine and tyrosine ( + 79.966) was also included as a variable modification. Searches were performed with full tryptic digestion and a maximum of 2 missed cleavages were allowed. The reverse database search option was enabled and all data was filtered to satisfy a false discovery rate (FDR) of 5%.

Data analyses were performed on log_2_ normalized abundances using an un-paired, two-tailed, heteroscedastic Student’s *t* test in Microsoft Excel. A 5% false discovery rate (FDR) threshold for each comparison was calculated using R Studio. For data visualization, principal component analysis (PCA) plots and volcano plots were generated using R Studio. Heatmaps were generated using GraphPad Prism 8. For overrepresentation analysis, significantly differentially expressed proteins (*P* < 0.05) were functionally categorized based on Reactome (https://reactome.org) via gene ontology (GO) classification using WebGestalt^26^ (http://www.webgestalt.org/). Gene ontology biological process was used as a functional database. Functional annotation of biological pathways was also performed using DAVID^27^ (https://david.ncifcrf.gov). WebGestalt and DAVID use the Benjamini-Hochberg Procedure to decrease the false discovery rate (FDR). Significantly enriched pathways are presented (FDR ≤ 0.05).

### Molecular modelling

The model for the IVS-1-1 mutant was built using Alphafold^66^. The 1VS-1-1 mutant model was then aligned with the β-globin proteins in the high-resolution X-ray structure of human hemoglobin (pdb code: 1A00^67^). The tetramer was relaxed by energy minimization with the Gromacs software^68^ using the Gromos 54A7 force-field^69^. The system was energy minimized with the steepest-descent method in order to remove excessive strain by performing 100 steps of minimization with harmonic restraints applied to all non-hydrogen atoms, followed by further 100 steps restraining the Cα atoms only, ending with 100 steps with no restraints.

### Statistical analysis

For statistical analysis of >2 groups, ANOVA with Tukey multiple comparison testing was performed. For analysis of 2 groups, a two-tailed Welch’s *t*-test was performed. In both cases, statistical tests were performed in GraphPad Prism 8. In all cases, measurements were taken from distinct samples.

### Reporting summary

Further information on research design is available in the Nature Portfolio Reporting Summary linked to this article.

### Supplementary information

Supplementary Information

Description of Additional Supplementary Files

Supplementary Data 1

Reporting Summary

### Source data

Source Data

## Data Availability

The proteomic data set is provided as a Supplementary Data 1, with raw files available through PRIDE (accession PXD044730). Source data are provided with this paper.
