# Supplementary material for: Human cellular model systems of β-thalassemia enable in-depth analysis of disease phenotype
Source: Nat Commun. 2023 Oct 6;14:6260. doi: 10.1038/s41467-023-41961-9 (PMC10558456; doi:10.1038/s41467-023-41961-9)

Human cellular model systems of β-thalassemia enable in-depth analysis of disease phenotype

Deborah E Daniels[1], Ivan Ferrer-Vicens[1], Joe Hawksworth[1], Tatyana N Andrienko[1], Elizabeth M Finnie[1], Natalie S Bretherton[1], Daniel C J Ferguson[1], A. Sofia F Oliveira[1], Jenn-Yeu A Szeto[1], Marieangela C Wilson[1], John N Brewin[2,3], Jan Frayne[1]*

[1]School of Biochemistry, University of Bristol, Bristol BS8 1TD, UK
[2]Haematology Department, King's college Hospital NHS Foundation, London, SE5 9RS, UK
[3]Red Cell Biology Group, Kings College London, London SE5 9NU, UK

*Correspondence should be addressed to: Jan.Frayne@Bristol.ac.uk

Supplementary Information

Supplementary Figure 1

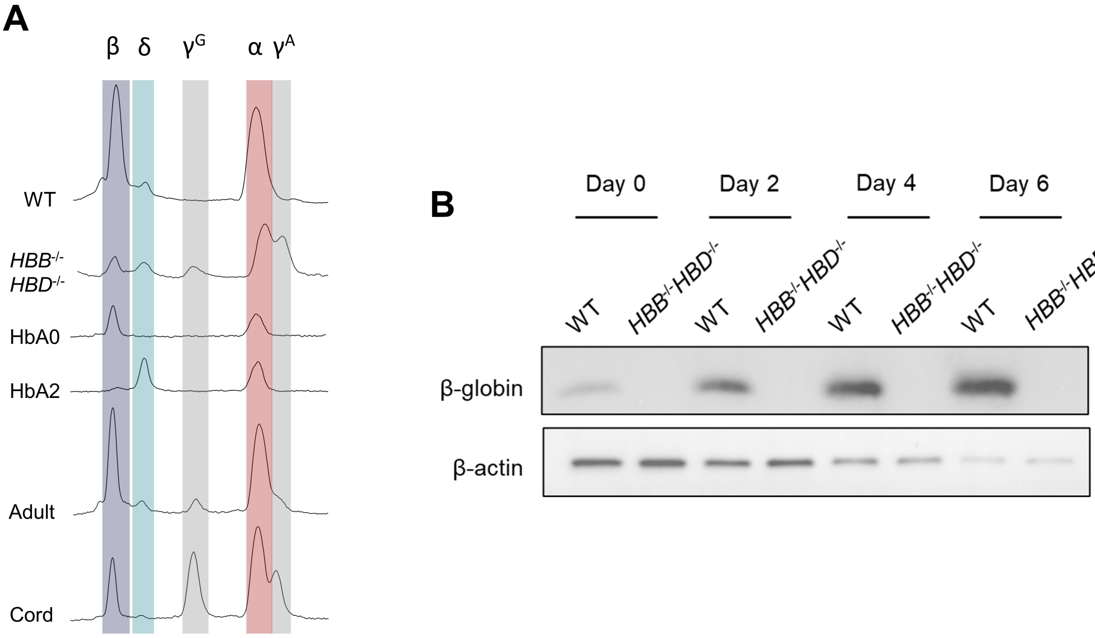

Supplementary Figure 1: Confirming loss of β-globin despite residual peak on RP-HPLC overlapping position of β-globin. These experiments were undertaken using a *HBB HBD* double knockout BEL-A line ($HBB^{-/-}HBD^{-/-}$) due to unavailability of a β-globin antibody that did not cross react with δ-globin. (A) RP-HPLC traces for WT and $HBB^{-/-}HBD^{-/-}$ at day 6 of differentiation showing presence of small residual peaks in the β-globin and δ-globin positions. Normal peak positions are identified for β-globin (β), δ-globin (δ), ^{G}γ-globin (^{G}γ), α-globin (α) and ^{A}γ-globin (^{A}γ). HbA0 and HbA2 purified controls, along with differentiated adult and cord blood samples, are included for reference. (B) Western blot of whole cell lysates from WT and $HBB^{-/-}HBD^{-/-}$ lines harvested at day 0, 2, 4 and 6 of differentiation, incubated with β-globin antibody, showing complete absence of β-globin in the $HBB^{-/-}HBD^{-/-}$ cells, and that the residual peak seen on RP-HPLC traces of the $HBB^{-/-}HBD^{-/-,}$ *and* $HBB^{-/-}$ lines is not β-globin; Mass Spectrometry reveal the peak to be due to other heme binding proteins. β-actin was used as a protein loading control.

Supplementary Figure 2

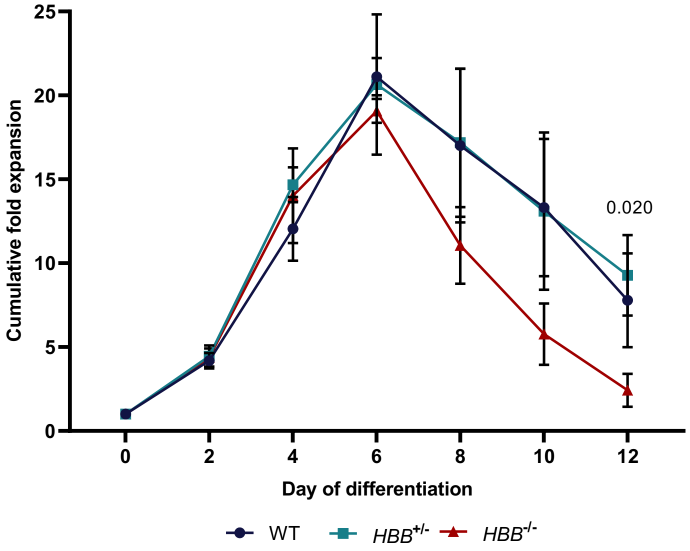

Supplementary Figure 2: Expansion curves of WT, *HBB*$^{+/-}$ and *HBB*$^{-/-}$ BEL-A during erythroid differentiation. Results show mean ± SD, n=3. *P*-values are marked where ANOVA testing is statistically significant. Source data are provided as a Source Data file.

Supplementary Figure 3

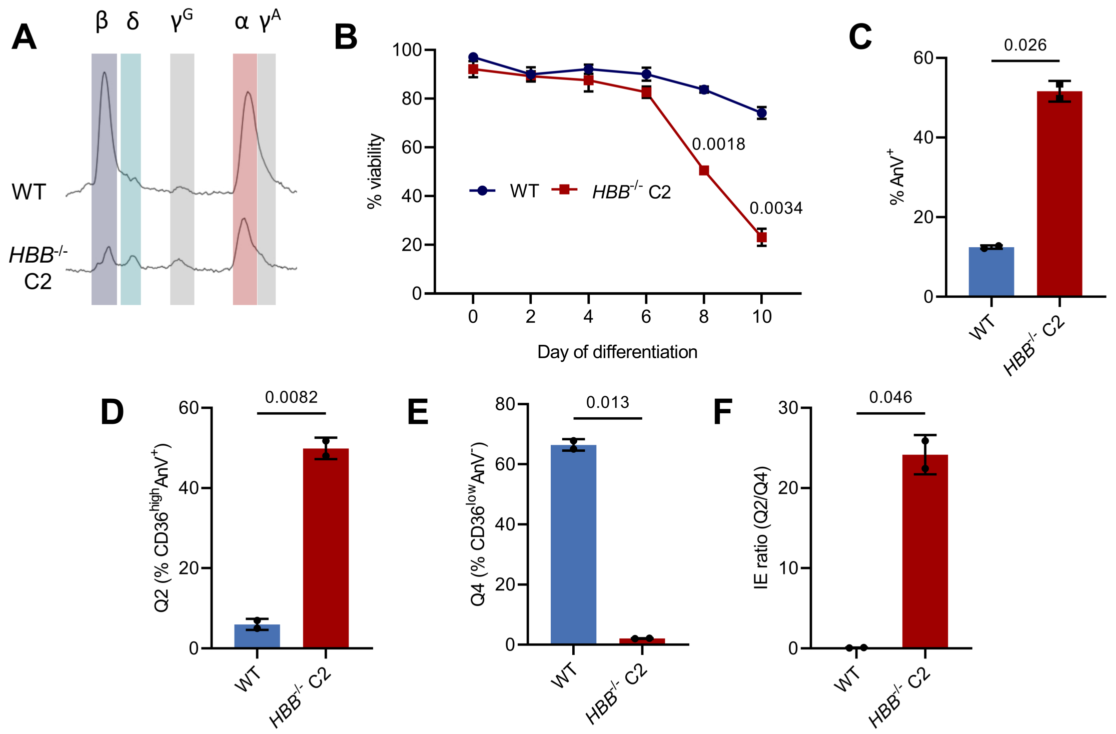

Supplementary Figure 3: An additional clonal *HBB*$^{-/-}$ BEL-A line demonstrates consistent phenotype. (A) RP-HPLC traces for WT and *HBB*$^{-/-}$ BEL-A at day 6 of differentiation. Peaks are identified for β-globin (β), δ-globin (δ), ^{G}γ-globin (^{G}γ), α-globin (α) and ^{A}γ-globin (^{A}γ). (B) Percentage viability during differentiation by trypan blue exclusion assay. (C) Percentage of Annexin V positive cells (AnV^{+}) in at day 7 of differentiation as determined by flow cytometry. Quantification of Q2 CD36^{high}AnV^{+} cells (D), Q4 CD36^{low}AnV^{-} cells (E) and IE ratio (Q2/Q4) (F) at day 7 of differentiation. Results show mean ± SD, n=2. *P*-values are marked where results of Welch's t-test are statistically significant. Source data are provided as a Source Data file.

Supplementary Figure 4

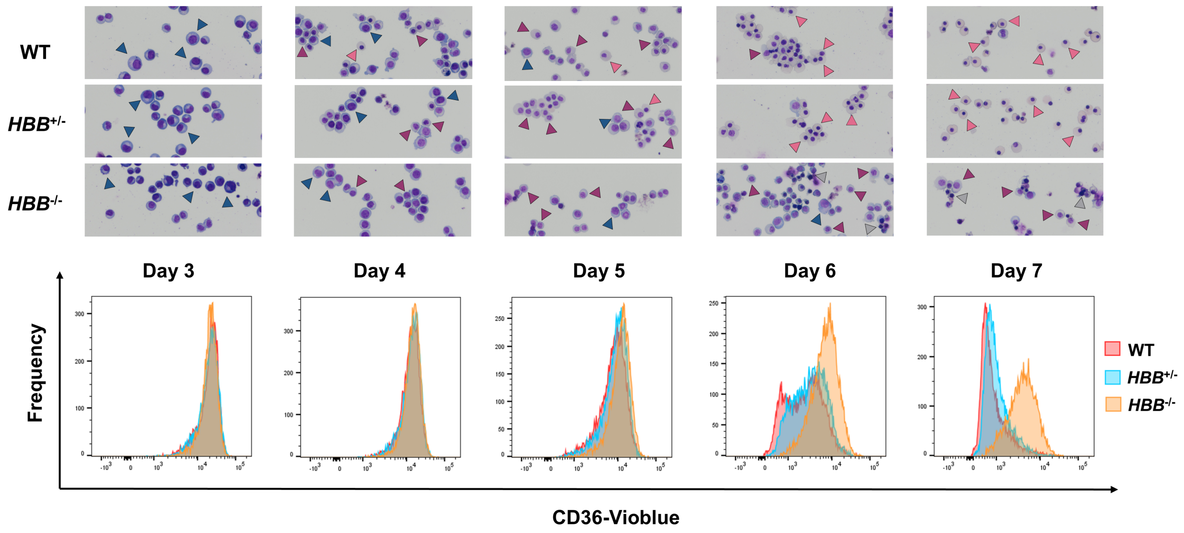

Supplementary Figure 4: Flow cytometry analysis of CD36 cell-surface abundance in WT, *HBB*$^{+/-}$ and *HBB*$^{-/-}$ BEL-A cells with corresponding cytospin images during erythroid differentiation. Arrowheads indicate the following cell types: blue, basophilic erythroblast; purple, polychromatic erythroblast; pink, orthochromatic erythroblast; grey, dead/apoptotic.

Supplementary Figure 5

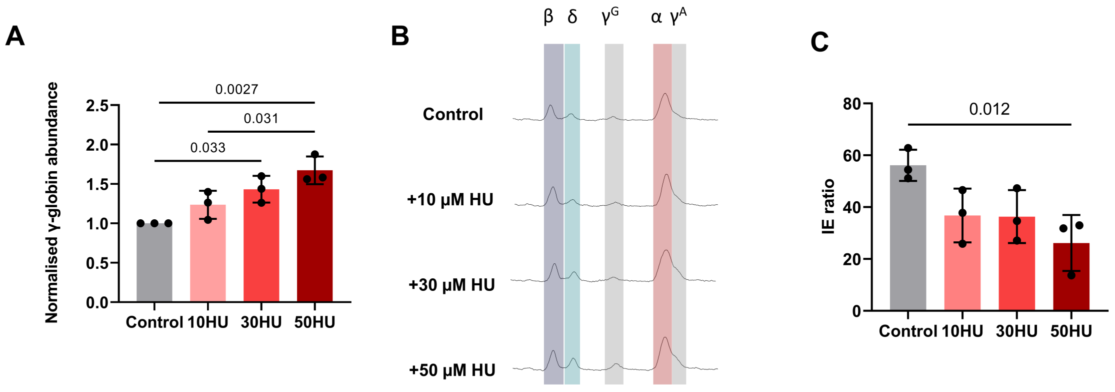

Supplementary Figure 5: *HBB*$^{-/-}$ cells respond to hydroxyurea (HU) to increase γ-globin expression and reduce ineffective erythropoiesis. Differentiated *HBB*$^{-/-}$ BEL-A cells were treated with 10, 30 or 50 μM of hydroxyurea (HU). (A) Abundance of total γ-globin (^{G}γ-globin + ^{A}γ-globin) at day 6 of differentiation quantified from RP-HPLC traces and normalised to total globin, shown as a proportion of control normalised γ-globin abundance. (B) Representative RP-HPLC traces. Peaks are identified for β-globin (β), δ-globin (δ), ^{G}γ-globin (^{G}γ), α-globin (α) and ^{A}γ-globin (^{A}γ). (C) Quantification of IE ratio at day 7 of differentiation from flow cytometry IE assay (%CD36^{high}AnV^{+}/ CD36^{low}AnV^{-}) for HU treatment of HBB$^{-/-}$ BEL-A cells. Results show mean ± SD, n=3. *P*-values are marked where results of ANOVA with Tukey multiple comparison testing are statistically significant. Source data are provided as a Source Data file.

Supplementary Figure 6

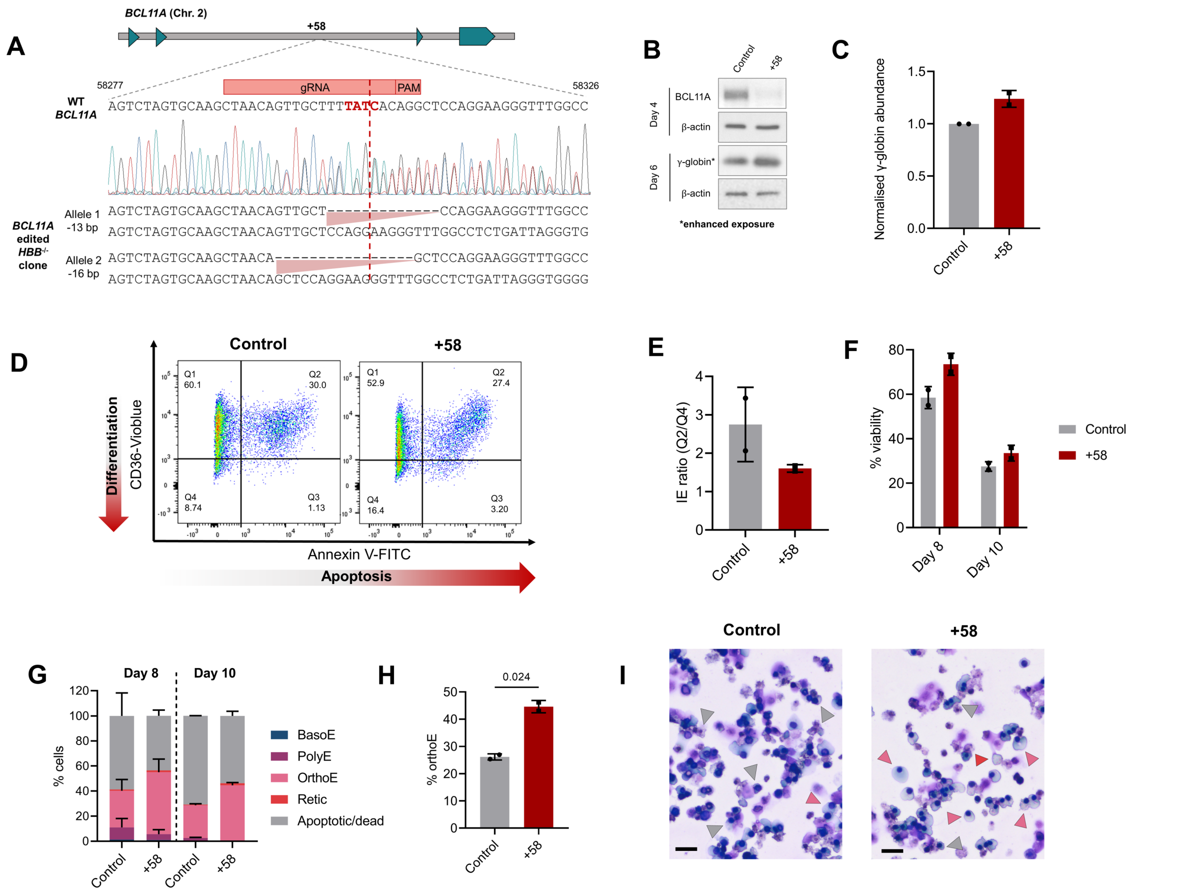

Supplementary Figure 6: CRISPR-Cas9 genome editing of the *BCL11A* +58 enhancer in *HBB*$^{-/-}$ BEL-A. (A) Schematic showing gDNA sequence analysis of *BCL11A* +58 enhancer edited HBB$^{-/-}$ BEL-A. Position of GATA box shown in red. (B) Representative western blots of control and +58 *BCL11A* enhancer edited *HBB*$^{-/-}$ BEL-A cell lysates harvested at day 4 and 6, incubated with BCL11A and γ-globin antibodies respectively. β-actin was used as a protein loading control (C) Abundance of total γ-globin (^{G}γ-globin + ^{A}γ-globin) at day 6 of differentiation quantified from RP-HPLC traces and normalised to total globin, shown as a proportion of control normalised γ-globin abundance. (D) Representative IE flow cytometry plots of control and +58 *BCL11A* enhancer edited *HBB*$^{-/-}$ BEL-A cells at day 7 and (E) Quantification of IE ratio (%CD36^{high}AnV^{+}/ CD36^{low}AnV^{-}). (F) Percentage viability of control and +58 *BCL11A* enhancer edited *HBB*$^{-/-}$ BEL-A during differentiation by trypan blue exclusion assay. (G) Morphology of control and +58 *BCL11A* enhancer edited *HBB*$^{-/-}$ BEL-A at day 8 and 10 of differentiation. (H) Percentage of orthochromatic erythroblasts at day 10 of differentiation. (I) Representative cytospin images at day 10 of differentiation. Arrowheads indicate the following cell types: polychromatic erythroblast; pink, orthochromatic erythroblast; red, reticulocyte; grey, dead/apoptotic. Scale bars 20 µm. Results show mean ± SD, n=2. *P*-values are marked where results of Welch's t-test are statistically significant. Source data are provided as a Source Data file.

Supplementary Figure 7

A

Peptide sequence	PSMs
MVHLTPEEK	11
SAVTALWGK	31
**VNVDEVGGEALGSLVSR**	**40**
LLVVYPWTQR	57
FFESFGDLSTPDAVMGNPK	71
VK	Not detected
AHGK	Not detected
KVLGAFSDGLAHLDNLK	6
GTFATLSELHCDK	14
LHVDPENFR	7
LLGNVLVCVLAHHFGK	12
EFTPPVQAAYQK	5
VVAGVANALAHKYH	20

B

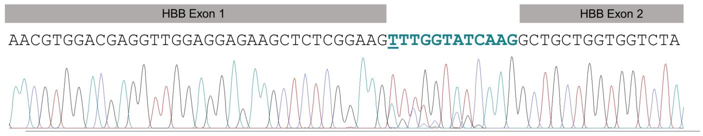

Supplementary Figure 7: Identification of IVS-1-1 β-globin splice variant. (A) Mass spectrometry results positively identify the predicted IVS-1-1 β-globin unique peptide sequence (bold) containing the additional 4 amino acids (red) from trypsin digest of unknown peak identified by RP-HPLC in Figure 3B. The number of peptide spectrum matches (PSMs) for each peptide from the trypsin digest of the variant β-globin are shown. (B) Chromatogram of IVS-1-1 BEL-A *HBB* transcript cDNA. Silent mutations resulting from the CRISPR edit are shown in red and the inserted 12 bases from the first intron of HBB as a result of splicing using the IVS-1-13 alternative splice site are shown in teal (with the G→T IVS-1-1 substitution underlined).

Supplementary Figure 8

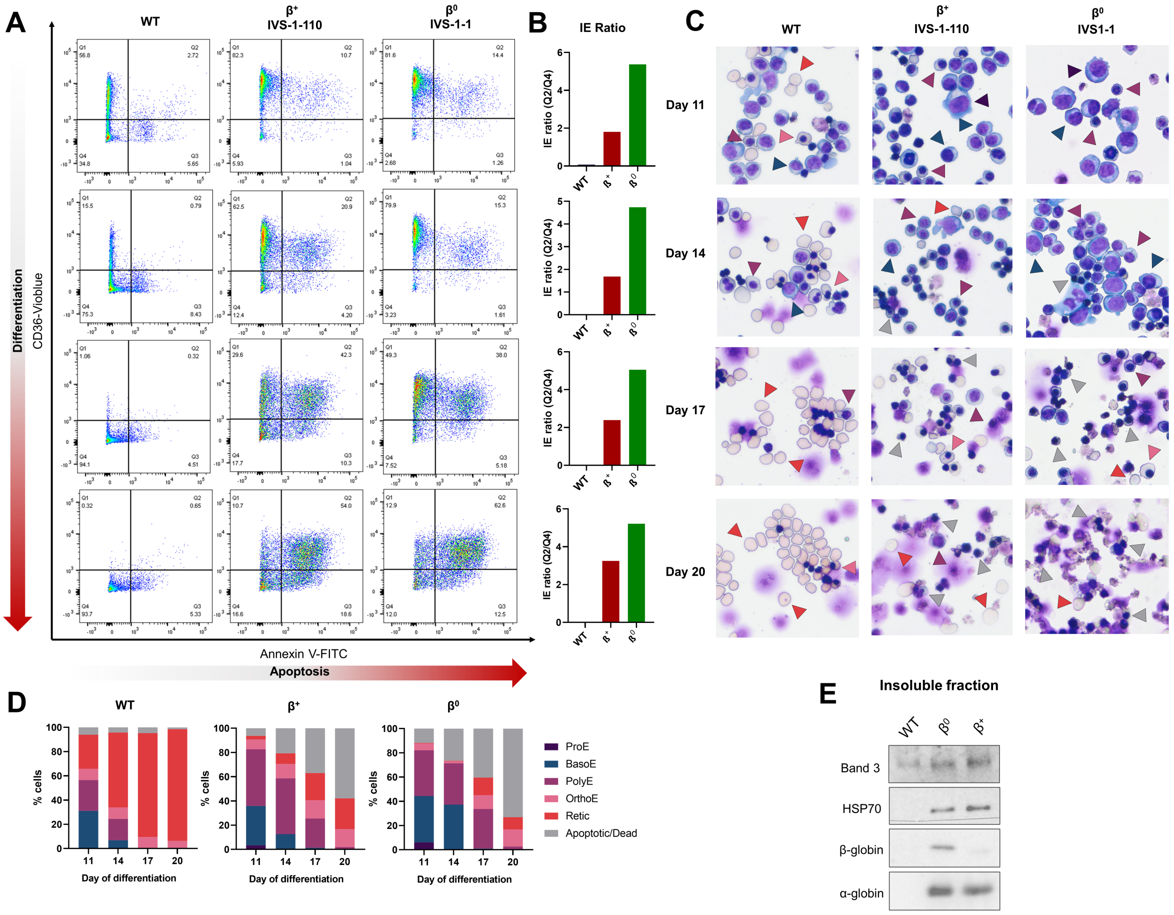

Supplementary Figure 8: Characterisation of cultured primary erythroblasts from β-thalassemia patient donors. Primary cultures were performed for two sets of patient cells (IVS-1-110 G→A [β^+] and IVS-1-1 G→T [β^0] alongside cells from healthy control individuals (control culture data shown is representative of three independent donors). (A) Representative IE flow cytometry plots at day 11, 14, 17 and 20 of differentiation. (B) Quantification of IE ratio (%$CD36^{high}AnV^+$/ $CD36^{low}AnV^-$). (C) Representative cytospin images of control and patient cell cultures. Arrowheads indicate the following cell types: dark purple, ProE; blue, BasoE; purple, PolyE; pink, OrthoE; red, Retic; grey, dead/apoptotic. (D) Morphology analysis of control and patient cell cultures. (E) Western blot of the insoluble fraction from control and patient cells harvested at day 15 of differentiation, incubated with α-globin, β-globin and HSP70 antibodies. Band 3 was used as a protein loading control. Source data are provided as a Source Data file.

Supplementary Figure 9

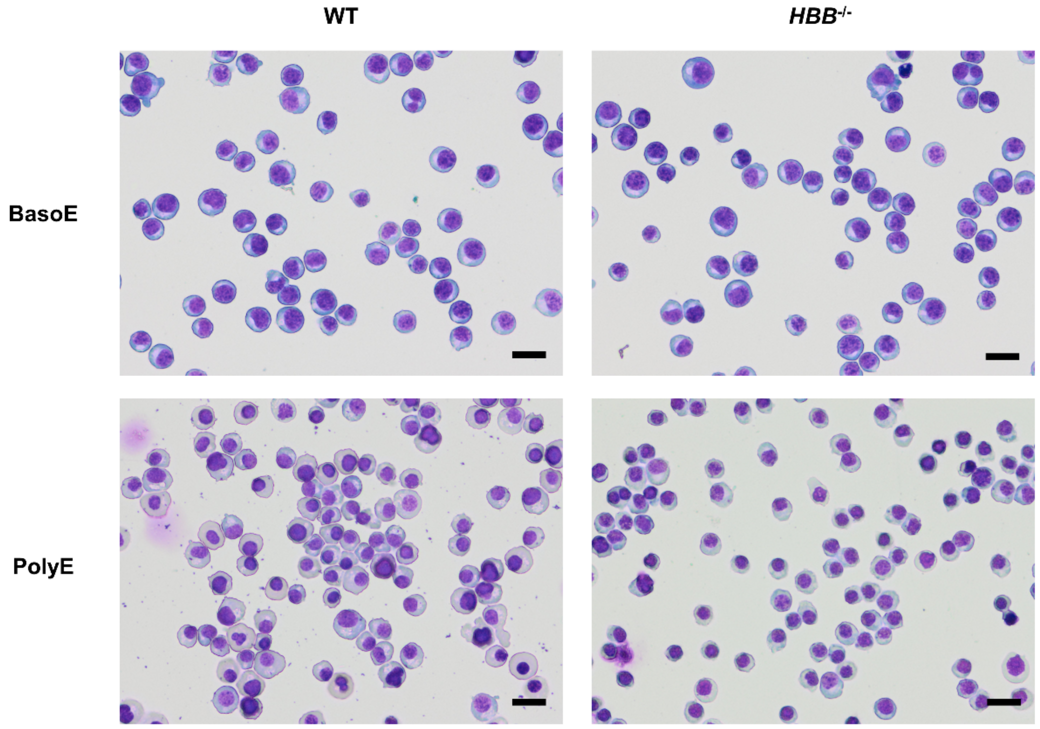

Supplementary Figure 9 Supplementary Figure 9: FACS isolated WT and *HBB*$^{-/-}$ cells for multiplex TMT-based comparative proteomics. Representative Leishman's stained cytospin images of basophilic (BasoE) and polychromatic (PolyE) erythroblasts after FACS isolation based on cell surface expression of CD36 and GPA. Scale bars 20 µm.

Supplementary Figure 10

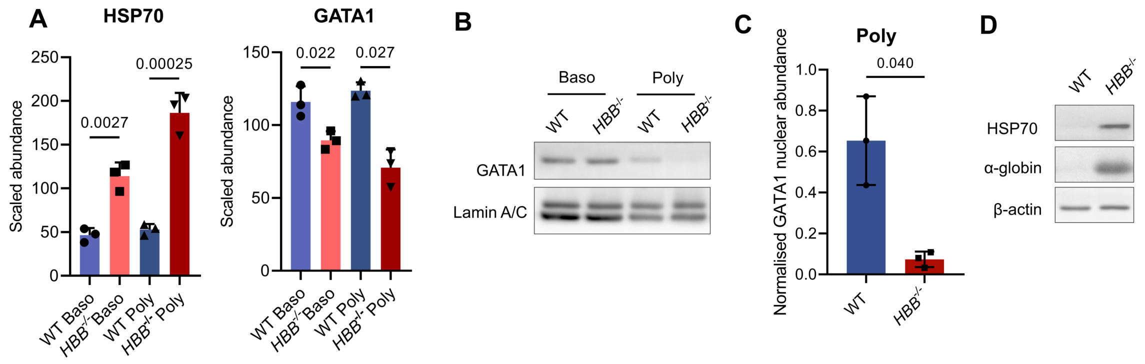

Supplementary Figure 10: HSP70 and GATA1 in β-thalassemia erythroid cells. (A) Scaled protein abundance of HSP70 and GATA1 in WT and *HBB*$^{-/-}$ cells from TMT-based comparative proteomic data. Data shown are abundance values normalized to total protein and scaled relative to 100. p values represent results from ANOVA performed on $\log_2$ normalized data. (B) Representative western blot of nuclear protein lysates from time points at which basophilic/polychromatic erythroblasts predominate (day2/5 for WT and day 3/6 for *HBB*$^{-/-}$ BEL-A) incubated with GATA-1 antibody. Lamin A/C is included as a protein loading control. (C) Densitometry quantification of GATA-1 nuclear abundance normalised to lamin A/C abundance. (D) Representative western blot of aggregate proteins from WT, and *HBB*$^{-/-}$ BEL-A harvested at day 6 of differentiation, incubated with α-globin and HSP70 antibodies. β-actin was used as a protein loading control. Results shown are mean ± SD, n=3. *P*-values represent of Welch's t-test results. Source data are provided as a Source Data file.

Supplementary Figure 11

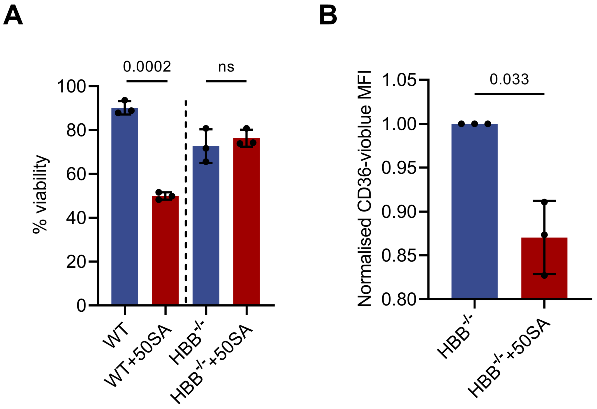

Supplementary Figure 11: Succinylacetone treatment of differentiating WT and *HBB*$^{-/-}$ cells. (A) Percentage viability of control and 50 μM succinylacetone treated (+50SA) WT and *HBB*$^{-/-}$ cells at day 7 of differentiation, analysed by trypan blue exclusion assay. (B) CD36-Vioblue median fluorescence intensity (MFI) at day 7 of differentiation normalised to untreated HBB$^{-/-}$ cells. Results show mean ± SD, n=3. *P*-values show results of Welch's t-test. ns = non-significant. Source data are provided as a Source Data file.

Supplementary Figure 12

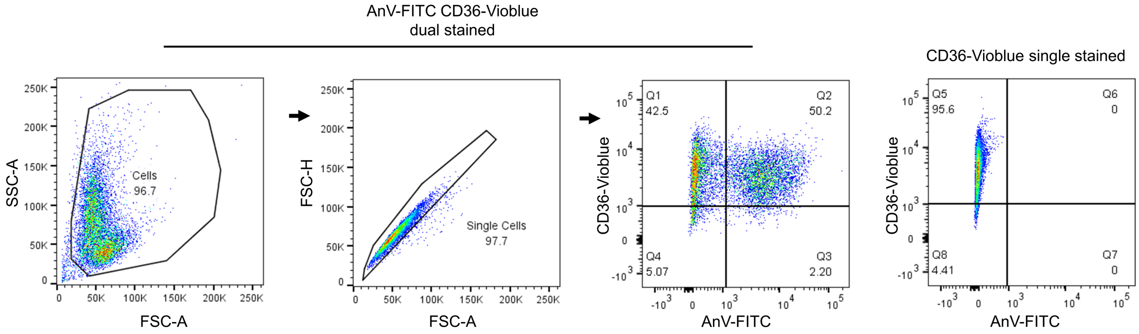

Supplementary Figure 12: Example IE flow cytometry gating strategy. A generous cell gate was used in order not to exclude potential apoptotic cells. Single cells were then gated by FSC-H vs FSC-A. The vertical quadrant divider for the IE plots was determined using a CD36-Vioblue only stained negative control for Annexin V (AnV) positive cells (right-hand plot). The horizontal quadrant divider was set at 10^3 as the chosen cut-off separating the $CD36^{high}$ and $CD36^{low}$ cell populations, allowing comparisons of cell maturation within each experiment.

Supplementary Table 1

Proteins (top 10) significantly increased or decreased in HBB$^{-/-}$ basophilic and polychromatic erythroid cells. *P* values represent results from 2-tailed heteroscedastic t-tests performed on $\log_2$ normalized data and are unadjusted for multiple comparisons (false discovery rate values are provided in source data). Source data are available via PRIDE (Accession PXD044730) and Supplementary Data 1.

			Basophilic HBB$^{-/-}$/WT		Polychromatic HBB$^{-/-}$/WT	
**Gene name**	**PSM**	**Unique Peptides**	**Fold difference**	***P*-value**	**Fold difference**	***P*-value**
NDRG1	4	3	4.75	$1.31x10^{-2}$	7.72	$2.14x10^{-4}$
ARG1	21	13	3.70	$2.25\ x10^{-3}$	4.72	$2.92x10^{-4}$
AKR1C3	21	7	3.10	$1.42\ x10^{-3}$	4.46	$1.13x10^{-3}$
SRXN1	15	7	4.08	$3.51\ x10^{-3}$	4.46	$1.95x10^{-3}$
HSPA1A	263	38	2.44	$2.67\ x10^{-3}$	3.50	$2.46x10^{-4}$
TOR3A	3	3	2.69	$1.27x10^{-2}$	3.50	$3.18x10^{-3}$
IL18	6	4	2.21	$1.54x10^{-2}$	3.50	$7.13x10^{-3}$
OTUD5	14	10	2.23	$3.63x10^{-4}$	3.09	$1.14x10^{-3}$
CTSB	12	6	1.74	$2.18x10^{-2}$	2.94	$7.92x10^{-3}$
XPO7	92	40	1.65	$2.84x10^{-2}$	2.90	$6.44x10^{-4}$
HBB	834	1	0.03	$3.79x10^{-5}$	0.01	$2.29x10^{-4}$
PHGDH	20	15	0.34	$6.75x10^{-2}$	0.09	$5.40x10^{-3}$
PSAT1	36	18	0.23	$9.63x10^{-4}$	0.11	$5.49x10^{-4}$
PACRGL	2	1	0.26	$3.77x10^{-4}$	0.11	$8.37x10^{-5}$
TMEM192	2	2	0.32	$1.15\ x10^{-3}$	0.15	$3.05x10^{-4}$
MCMBP	36	9	0.55	$3.97\ x10^{-3}$	0.16	$6.08x10^{-4}$
ZNF648	49	1	0.26	$1.22x10^{-4}$	0.16	$1.23x10^{-4}$
ASNS	32	2	0.31	$1.82x10^{-2}$	0.16	$1.84x10^{-3}$
INF2	15	13	0.37	$3.55\ x10^{-3}$	0.17	$1.24x10^{-3}$
LEPR	7	5	0.31	$2.60\ x10^{-3}$	0.17	$2.97x10^{-3}$

Supplementary Table 2

Proteins with 2-fold or more significantly increased phosphorylation in HBB$^{-/-}$ polychromatic erythroid cells. *P* values represent results from 2-tailed heteroscedastic t-tests performed on $\log_2$ normalized data and are unadjusted for multiple comparisons (false discovery rate values are provided in source data). Source data are available via PRIDE (Accession PXD044730).

Gene name	HBB$^{-/-}$/WT Fold difference	*P*-value	Peptide Sequence	Phosphosites
TMEM192	10.70	2.14×10^{-04}	TISSLEEIVEK	Ambiguous
RAB8A	8.39	2.51×10^{-04}	NIEEHASADVEK	S7 (Phospho)
ALS2	6.40	1.51×10^{-02}	LSLPGLLSQVSPR	S11 (Phospho)
PACRGL	6.27	9.79×10^{-03}	SSLSTSSPESAR	Ambiguous
MCMBP	5.06	6.93×10^{-03}	VHSPPASLVPR	S3 (Phospho)
WDR44	4.89	1.16×10^{-02}	LTQTSSTEQLNVLETETEVLNK	S6 (Phospho)
SUB1	4.69	4.97×10^{-02}	ELVSSSSSGSDSDSEVDKK	S12 (Phospho)
CARS1	4.40	2.5×10^{-03}	ALQEGEGDLSISADR	S12 (Phospho)
LYN	4.00	4.16×10^{-02}	ASSPSPR	S2, S5 (Phospho)
COP53	3.97	2.92×10^{-02}	SMGSQEDDSGNKPSSYS	S1, S4, S9 (Phospho)
OSBP2	2.96	1.71×10^{-02}	VMNTHSDDSGDDDEATTPADK	S9(Phospho)
WDR3	2.80	4.81×10^{-02}	GSSPGIQDTLEAEDGAFETDEAPEDR	S3(Phospho)
ESPN	2.73	1.80×10^{-02}	ELPPPPPPPPPLPEAASSPPPAPPLPLESAGPGCGQR	Ambiguous
C7orf50	2.60	2.09×10^{-02}	ELDEEGSDPPLPGR	S7 (Phospho)
H2AX	2.47	8.83×10^{-03}	ATQASQEY	S5 (Phospho)
YWHAE	2.44	9.66×10^{-05}	AAFDDAIAELDTLSEESYK	S14 (Phospho)
RGCC	2.42	2.61×10^{-02}	SSASVSDSSGFSDSESADSLYR	S9 (Phospho)
MSN	2.14	4.67×10^{-02}	IDEFESM	S6 (Phospho)
SLC29A1	2.11	1.35×10^{-02}	EESGVSVSNSQPTNESHSIK	S18 (Phospho)
JMJD1C	2.11	1.32×10^{-02}	VDLTQSSVTNASSGNDHLNMEKEK	T9 (Phospho)
DUS3L	2.08	2.96×10^{-02}	RFSQGPTPAAAVPEGTAAEGAPR	S3 (Phospho)
STX4	2.02	2.30×10^{-02}	QGDDSSDEEDKER	S5, S6 (Phospho)

Uncropped western blot images for Supplementary Data

Figure S1B

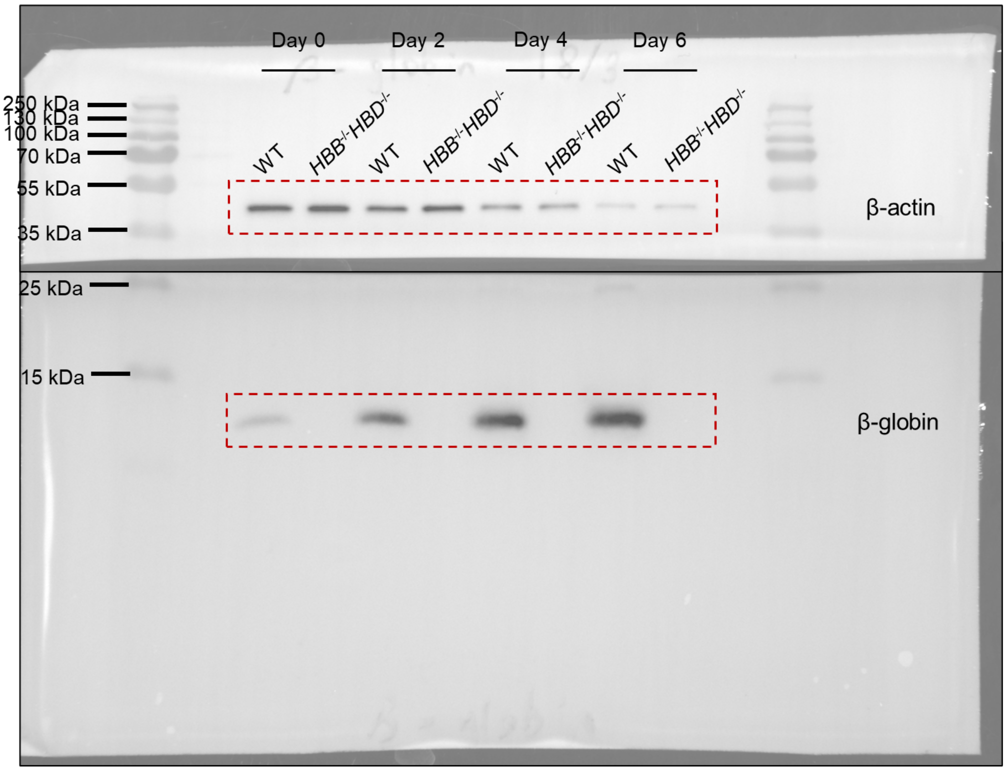

Figure S6B upper

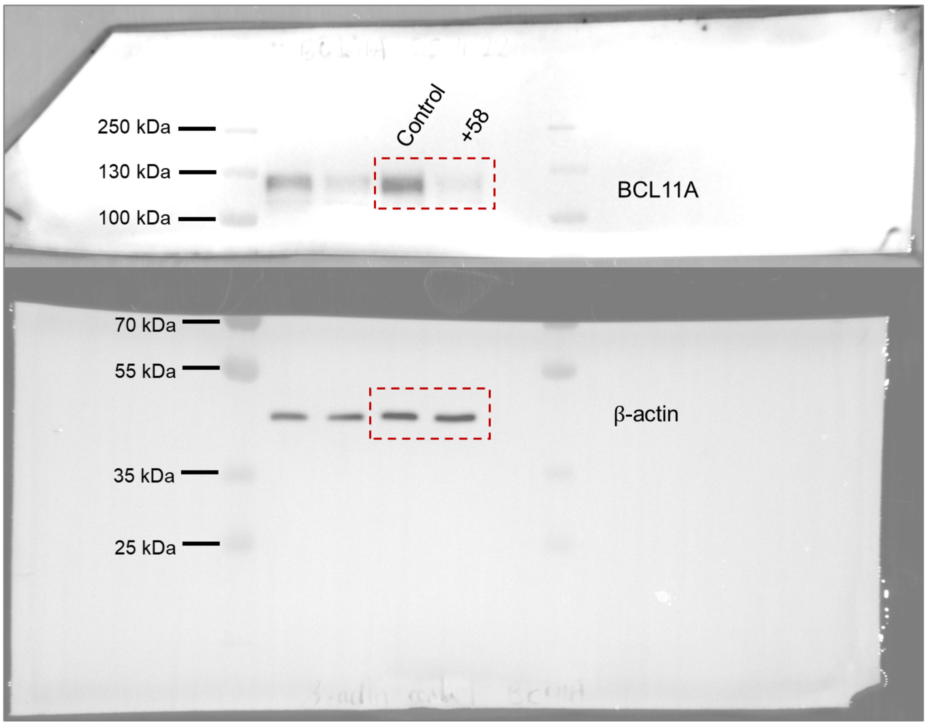

Figure S6B lower

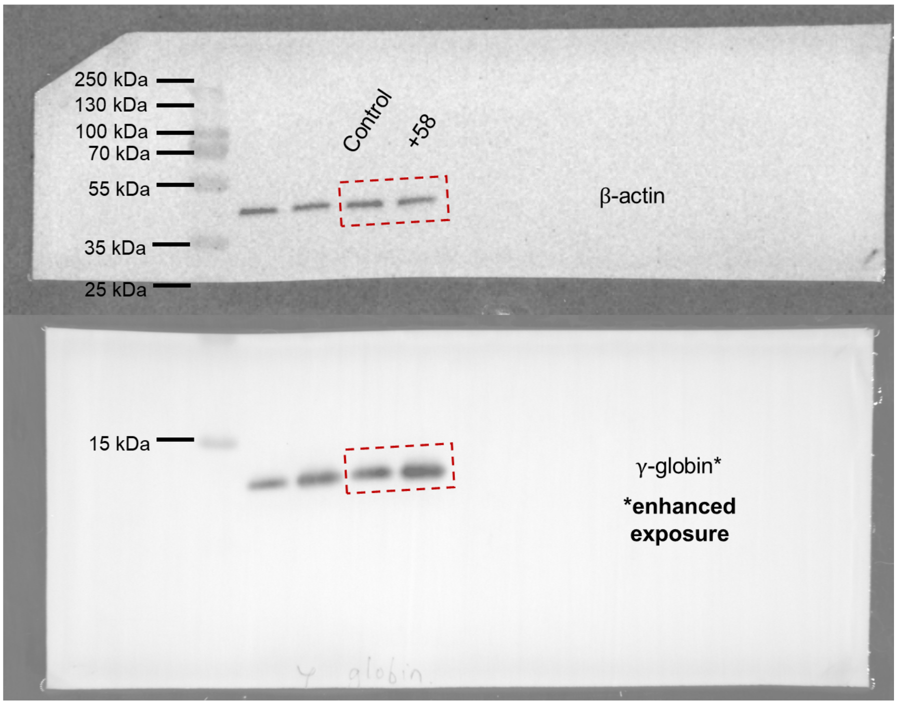

Figure S8E

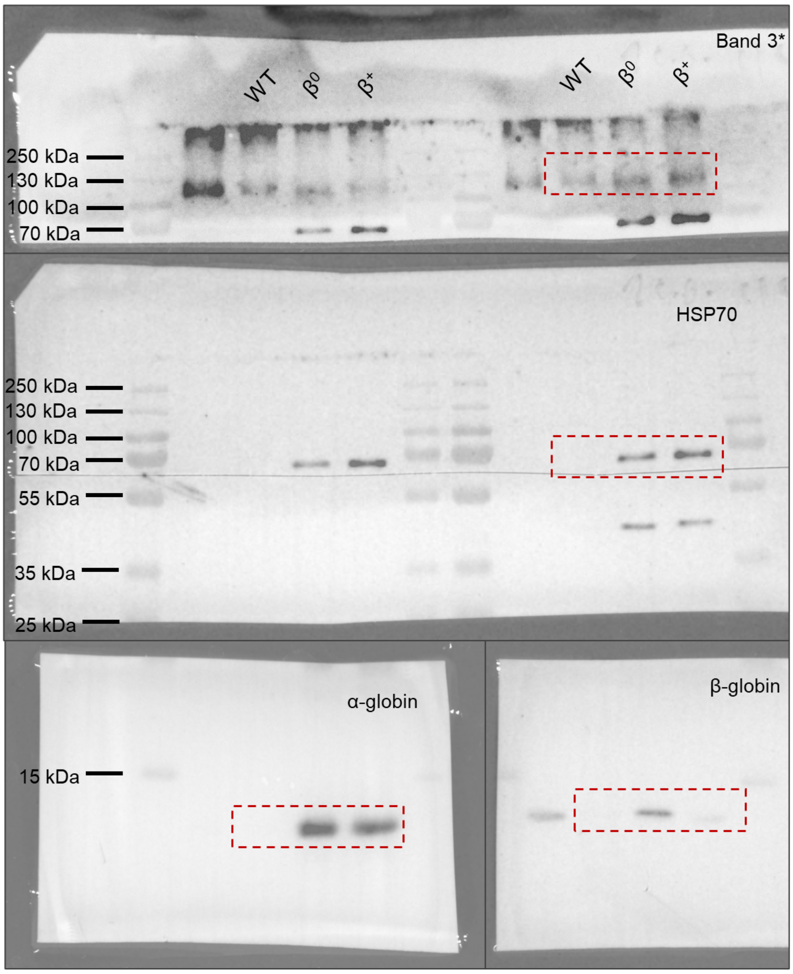

*Re-probed after HSP70

Figure S10B

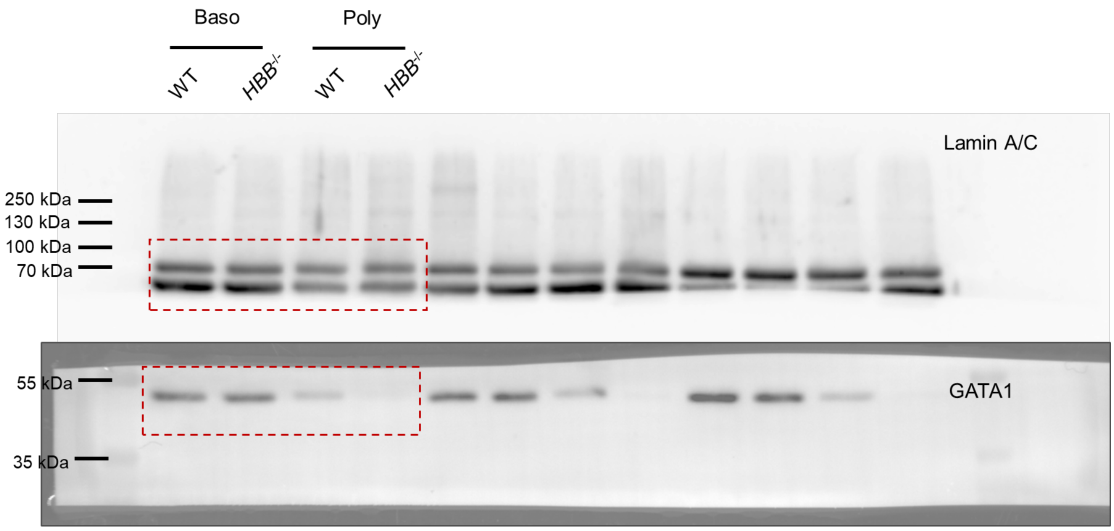

Figure S10D

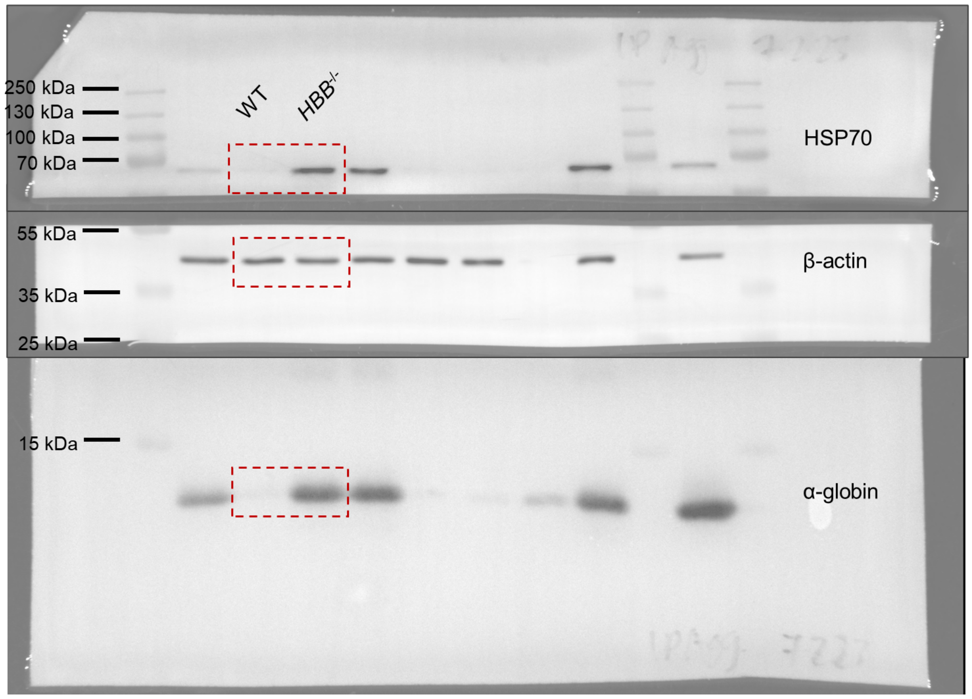

Supplement: Supplementary file 1 — Supplementary Information [file 41467_2023_41961_MOESM1_ESM.pdf]
