# Supplementary material for: Human cellular model systems of β-thalassemia enable in-depth analysis of disease phenotype
Source: Nat Commun. 2023 Oct 6;14:6260. doi: 10.1038/s41467-023-41961-9 (PMC10558456; doi:10.1038/s41467-023-41961-9)
Supplement: Supplementary file 2 — Description of Additional Supplementary Files [file 41467_2023_41961_MOESM2_ESM.pdf]

Description of Additional Supplementary Files

Supplementary Data 1: Processed proteomic data
