# Supplementary material for: Human cellular model systems of β-thalassemia enable in-depth analysis of disease phenotype
Source: Nat Commun. 2023 Oct 6;14:6260. doi: 10.1038/s41467-023-41961-9 (PMC10558456; doi:10.1038/s41467-023-41961-9)
Supplement: Supplementary file 4 — Reporting Summary [file 41467_2023_41961_MOESM4_ESM.pdf]

nature portfolio

Corresponding author(s): Jan Frayne

Last updated by author(s): Aug 22, 2023

Reporting Summary

Nature Portfolio wishes to improve the reproducibility of the work that we publish. This form provides structure for consistency and transparency in reporting. For further information on Nature Portfolio policies, see our Editorial Policies and the Editorial Policy Checklist.

Statistics

For all statistical analyses, confirm that the following items are present in the figure legend, table legend, main text, or Methods section.

n/a	Confirmed	
☐	☒	The exact sample size (*n*) for each experimental group/condition, given as a discrete number and unit of measurement
☐	☒	A statement on whether measurements were taken from distinct samples or whether the same sample was measured repeatedly
☐	☒	The statistical test(s) used AND whether they are one- or two-sided<br>*Only common tests should be described solely by name; describe more complex techniques in the Methods section.*
☒	☐	A description of all covariates tested
☒	☐	A description of any assumptions or corrections, such as tests of normality and adjustment for multiple comparisons
☐	☒	A full description of the statistical parameters including central tendency (e.g. means) or other basic estimates (e.g. regression coefficient) AND variation (e.g. standard deviation) or associated estimates of uncertainty (e.g. confidence intervals)
☐	☒	For null hypothesis testing, the test statistic (e.g. *F*, *t*, *r*) with confidence intervals, effect sizes, degrees of freedom and *P* value noted<br>*Give P values as exact values whenever suitable.*
☒	☐	For Bayesian analysis, information on the choice of priors and Markov chain Monte Carlo settings
☒	☐	For hierarchical and complex designs, identification of the appropriate level for tests and full reporting of outcomes
☒	☐	Estimates of effect sizes (e.g. Cohen's *d*, Pearson's *r*), indicating how they were calculated

Our web collection on statistics for biologists contains articles on many of the points above.

Software and code

Policy information about availability of computer code

Data collection	For proteomic analyses, all spectra were acquired using an Orbitrap Fusion Tribrid mass spectrometer controlled by Xcalibur 2.1 software (Thermo Scientific). For flow cytometer Diva software version 8.0.2 and 8.0.3 were used.
Data analysis	Flow cytometry data was analysed using FlowJo v10.6.1 (FlowJoLLC). Sequencing data was analysed using TIDE (Brinkman et al Nucleic Acids Res 2014;42,e168) and TIDER (Brinkman et al Nucleic Acids Res 2018;46,e58) web tools (https://tide.nki.nl/). The model forthe IVS 1-1 mutant was built using Alphafold (Jumper et Nature 2021:596,583-589). The hemoglobin tetramer was analysed using Gromacs software (Abraham et al Software 2015;1-2,19-25) using the Gromos 54A7 force-field (Schmid et al Eur Biophys J 2011:40,843-856). For proteomics the raw data files were processed and quantified using Proteome Discoverer software v2.4 (Thermo Scientific) and searched against the UniProt Human database (downloaded January 2022: 178,486 entries) using the SEQUEST algorithm.

For manuscripts utilizing custom algorithms or software that are central to the research but not yet described in published literature, software must be made available to editors and reviewers. We strongly encourage code deposition in a community repository (e.g. GitHub). See the Nature Portfolio guidelines for submitting code & software for further information.

Data

Policy information about availability of data

All manuscripts must include a data availability statement. This statement should provide the following information, where applicable:

- Accession codes, unique identifiers, or web links for publicly available datasets
- A description of any restrictions on data availability
- For clinical datasets or third party data, please ensure that the statement adheres to our policy

The proteomic data generated in this study have been deposited in the PRIDE database under accession code PXD044730, and the processed data uploaded as a Supplementary Data File. Data underlying the graphs presented in each figure are provided in the Source Data file.

Research involving human participants, their data, or biological material

Policy information about studies with human participants or human data. See also policy information about sex, gender (identity/presentation), and sexual orientation and race, ethnicity and racism.

Reporting on sex and gender	Fully anonymised samples of peripheral blood mononuclear cells were obtained as a waste product from donor blood of three independent donors, and from blood samples of two beta thalassemia patients. As the ethics stipulates use of anonymised samples, it was not possible to report on sex and gender in this study.
Reporting on race, ethnicity, or other socially relevant groupings	*Please specify the socially constructed or socially relevant categorization variable(s) used in your manuscript and explain why they were used. Please note that such variables should not be used as proxies for other socially constructed/relevant variables (for example, race or ethnicity should not be used as a proxy for socioeconomic status).*<br>*Provide clear definitions of the relevant terms used, how they were provided (by the participants/respondents, the researchers, or third parties), and the method(s) used to classify people into the different categories (e.g. self-report, census or administrative data, social media data, etc.)*<br>*Please provide details about how you controlled for confounding variables in your analyses.*
Population characteristics	Blood donors can donate once they have reached their 17th birthday. 1st time donors can donate up to their sixty-sixth birthday. Regular donors (i.e. those who give at least one donation in a two year period) can continue to donate beyond 70 years, provided they remain otherwise fit and well. The overall donor base is also more balanced towards women. At the end of November 2019, NHSBT had 818,831 active donors, of whom 354,345 (43%) were male and 464,486 (57%) female. In UK largest proportion of donors are Caucasian<br>The beta thalassmeia patients were both adults but further information is not available due to the samples being full anonymised. Beta thalassemia is not a sex linked disorder. Beta thalassemia most commonly affects people who are of Mediterranean (Greek, Italian and Middle Eastern) or Asian descent. It is also relatively common in people of African descent.
Recruitment	Recruitment for blood donors is performed by NHSBT. The beta thalassemia patients were recruited by their treating clinician based purely on availability of patients attending for blood transfusion and patient consent.
Ethics oversight	Adult CD34+ cells were isolated from LRS cones, with informed consent from all donor, and used in accordance with the Declaration of Helsinki and approved by the National Health Service Ethics Committee (reference number 08/H0102/06) and the Bristol Research Ethics Committee (reference 12/SW/0199). Whole blood from beta thalassemia patients was obtained under ethics board North of Scotland REC (18/NS/0005) with informed consent.

Note that full information on the approval of the study protocol must also be provided in the manuscript.

Field-specific reporting

Please select the one below that is the best fit for your research. If you are not sure, read the appropriate sections before making your selection.

☒ Life sciences ☐ Behavioural & social sciences ☐ Ecological, evolutionary & environmental sciences

For a reference copy of the document with all sections, see nature.com/documents/nr-reporting-summary-flat.pdf

Life sciences study design

All studies must disclose on these points even when the disclosure is negative.

Sample size	No sample size calculation was performed. Sample sizes were based on previous studies and included at least three replicates.
Data exclusions	There have been no data samples excluded.
Replication	At least three replicates were analysed per group. All attempts at replication were successful.
Randomization	There was no randomization since replicate groups were chosen according to their phenotype.

Blinding | The investigators of this study were not blinded for analysis of flow cytometry data as the same gating was used for all groups. For microscopy, slides were counted blindly.

Reporting for specific materials, systems and methods

We require information from authors about some types of materials, experimental systems and methods used in many studies. Here, indicate whether each material, system or method listed is relevant to your study. If you are not sure if a list item applies to your research, read the appropriate section before selecting a response.

Materials & experimental systems

n/a	Involved in the study
☐	☒ Antibodies
☐	☒ Eukaryotic cell lines
☒	☐ Palaeontology and archaeology
☒	☐ Animals and other organisms
☒	☐ Clinical data
☒	☐ Dual use research of concern
☒	☐ Plants

Methods

n/a	Involved in the study
☒	☐ ChIP-seq
☐	☒ Flow cytometry
☒	☐ MRI-based neuroimaging

Antibodies

Antibodies used

Antibodies used for flow cytometry were: Mouse anti-CD36-Vioblue conjugated antibody (Clone AC106, Cat. No. 130-095-482, Lot No. 5220209840, Miltenyi Biotec; dilution 1:11), Annexin V-FITC conjugated antibody (Cat. No. 130-093-060, Lot No. 5220106164, 5230406604, Miltenyi Biotec; dilution 1:10).

Primary antibodies used were: Monoclonal mouse anti-hemoglobin α (Clone D-4, Cat. No. sc-514378, Lot No. I0916, Santa Cruz; dilution 1:3000), monoclonal mouse anti-hemoglobin β (Clone 37-8, Cat. No. sc-21757, Lot No. G2418, Santa Cruz; dilution 1:3000), monoclonal mouse anti-hemoglobin γ-globin (Clone 51-7, Cat. No. sc-21756, Lot No. G0318, Santa Cruz; dilution 1:1500), monoclonal rat anti-GATA1 (Clone N1, Cat. No. sc-266, Lot No. E1713, Santa Cruz; dilution 1:1500), polyclonal rabbit anti-Lamin A/C (Clone H-110, Cat. No. sc-20681, Lot No. B0111, Santa Cruz; dilution 1:1000), monoclonal mouse anti-β-actin (AC-15, Cat. No. A1978, Batch No. 0000086303, Merck; dilution 1:3000), monoclonal mouse anti-Ctip1/BCL-11A (Clone 14B5, Cat. No. ab19487, Lot No. GR3383959-1, Abcam; dilution 1:2000), mouse anti-CD233/Band3 (Clone BRIC 170, IBGRL; dilution 1:500) mouse anti-Glycophorin A (Clone BRIC 256; IBGRL; dilution 1:1), polyclonal rabbit anti-HSP70 (Cat. No. Cat. No. ADI-SPA-812-D, Lot No. 09011646, Enzo Life Sciences; dilution 1:1000).

Secondary antibodies used were: Polyclonal rabbit anti-mouse immunoglobulins-HRP conjugated (Cat. No. P0260, Lot No. 41424309, DAKO; dilution 1:3000), polyclonal swine anti-rabbit immunoglobulins-HRP conjugated (Cat. No. P0399, Lot No. 41270083, DAKO; dilution 1:3000), polyclonal goat anti-rat immunoglobulins-HRP conjugated (Cat. No. 112-035-003, Jackson ImmunoResearch; dilution 1:10000).

Validation

Validations for the antibodies used for flow cytometry: Mouse anti-CD36-Vioblue conjugated antibody (Clone AC106, Cat. No. 130-095-482, Miltenyi Biotec) is extensively validated by the vendor (Specificity and sensitivity assay tests) (https://www.miltenyibiotec.com/GB-en/products/cd36-antibody-anti-human-ac106.html#conjugate=viogreen:size=100-tests-in-1-ml). Annexin V-FITC conjugated antibody (Cat. No. 130-093-060, Miltenyi Biotec) was validated by the vendor where Jurkat cells, cultured with staurosporine (50 nM) for 15 hours, were stained with Annexin V conjugates followed by staining with DAPI and analyzed by flow cytometry (https://www.miltenyibiotec.com/GB-en/products/annexin-v-conjugates.html#conjugate=fitc:size=100-tests-in-1-ml).

Validations for the primary antibodies used: Monoclonal mouse anti-hemoglobin α (Clone D-4, Cat. No. sc-514378, Santa Cruz) was validated by the vendor using Western blot analysis on K-562, TF-1, MEG-01 and HEL 92.1.7 cell lines and human peripheral blood whole cell lysates (https://www.scbt.com/p/hemoglobin-alpha-antibody-d-4). Monoclonal mouse anti-hemoglobin β (Clone 37-8, Cat. No. sc-21757, Santa Cruz) was validated by the vendor using Western blot analysis in TF-1 and HEL 92.1.7 cell lines and human peripheral blood whole cell lysates, and also for flow cytometry analysis of fixed and permeabilized human red blood cells (https://www.scbt.com/p/hemoglobin-beta-antibody-37-8). Monoclonal mouse anti-hemoglobin γ-globin (Clone 51-7, Cat. No. sc-21756, Santa Cruz) was validated by the vendor using Western blot analysis on TF-1, HEL 92.1.7 and MEG-01 cell lines and human peripheral blood whole cell lysates and human placenta tissue extracts. The antibody was also validated using flow cytometry analysis of fixed and permeabilized human red blood cells (https://www.scbt.com/p/hemoglobin-gamma-antibody-51-7). Monoclonal rat anti-GATA1 (Clone N1, Cat. No. sc-266, Santa Cruz) was validated by the vendor using Western blot analysis of GATA-1 expression in K-562 nuclear extract or in HEL 92.1.7, MEG-01 and TF-1 whole cell lysates (https://www.scbt.com/p/gata-1-antibody-n1). Polyclonal rabbit anti-Lamin A/C (Clone H-110, Cat. No. sc-20681, Santa Cruz) was validated by the vendor using Western blot analysis of Lamin A/C expression in WI 38 and Hs68 whole cell lysates. It was also validated by immunofluorescence staining of methanol-fixed C32 cells showing nuclear envelope localization (https://datasheets.scbt.com/sc-20681.pdf). For monoclonal mouse anti-β-actin (Clone AC-15, Cat. No. A1978, Merck) the vendor shows images collected from different publications where the antibody was validated by Western blot, immunofluorescence and immunohistochemistry (https://www.sigmaaldrich.com/GB/en/product/sigma/a1978). Monoclonal mouse anti-Ctip1/BCL-11A (Clone 14B5, Cat. No. ab19487, Abcam) was validated by the vendor using Western blot analysis in HAP1, Raji and Jurkat Cell Lines. No band was observed when BCL11A (Ctip1) knockout cells were examined. The antibody was also validated using flow cytometry analysis of fixed and permeabilized Ramos cells (https://www.abcam.com/products/primary-antibodies/ctip1bcl-11a-antibody-14b5-ab19487.html). Mouse anti-CD233/Band3 (Clone BRIC 170, IBGRL) has been validated by IBGRL and it has been used in different publications (https://nhsbtdbe.blob.core.windows.net/umbraco-assets-corp/6623/

bric-170.pdf). Polyclonal rabbit anti-HSP70 (Cat. No. ADI-SPA-812, Enzo Life Sciences) was validated by the vendor using Western blot analysis of recombinant human HSP70/HSP72 and PC12 and HeLa cell lines (control and heat shocked)(https://www.enzolifesciences.com/ADI-SPA-812/hsp70-hsp72-polyclonal-antibody/).

The different links provided above contain a list of relevant citations for each antibody.

Eukaryotic cell lines

Policy information about cell lines and Sex and Gender in Research

Cell line source(s)	The BEL-A cell line was made in Prof Jan Fraynes lab from adult bone marrow CD34+ cells obtained from Stem Cell Technologies. Creation of the line is described in Trakarnsanga et al Nat Comm 2017 14;8:14750.
Authentication	Authentification of the line is described in Trakarnsanga et al Nat Comm 2017 14;8:14750. This included morphological and flow cytometry analysis of key red blood cell membrane proteins during differentiation in comparison with primary adult erythroid cells, comparative proteomic analysis (TMT-labelling) and deformability analysis of BEL-A compared to primary adult reticulocytes, oxygen binding and release of BEL-A compared to primary adult erythroid cells, karyotype analysis.
Mycoplasma contamination	BEL-A tested negative for mycoplasm. All lines made in study are derived from BEL-A
Commonly misidentified lines (See ICLAC register)	There are no commonly misidentified lines used in this study.

Flow Cytometry

Plots

Confirm that:

☒ The axis labels state the marker and fluorochrome used (e.g. CD4-FITC).

☒ The axis scales are clearly visible. Include numbers along axes only for bottom left plot of group (a 'group' is an analysis of identical markers).

☒ All plots are contour plots with outliers or pseudocolor plots.

☒ A numerical value for number of cells or percentage (with statistics) is provided.

Methodology

Sample preparation	Aliquots of 2-3x10*5 cells were incubated with CD36-Vioblue conjugated antibody (130-095-482; Milenyi) in PBS containing 1% (w/v) BSA (Park Scientific Ltd)and 2mg/ml glucose (PBS-AG), followed by incubation with Annexin V-FITC (130-093-060; Miltenyi) or Annexin V-APC (640920, Biolegend) conjugated antibody in Annexin V binding buffer (10mM Hepes, pH 7.4; 140mM NaCL; 2.5mM CaCL2).
Instrument	BD LSR Fortessa flow cytometer
Software	FlowJo v10.6.1 (FlowJo LLC)
Cell population abundance	No cell population sorting carried out.
Gating strategy	The abundance of membrane protein CD36, which decreases during erythroid cell differentiation, was first evaluated. CD36 levels decreased from day 6 of differentiation in WT and HBB+/- cultures, but was retained in HBB-/- cultures, correlating with arrested differentiation of the HBB-/- cells at the polychromatic stage (Supplementary Figure 4), as determined by morphological analysis, and provided effective resolution of transition from polychromatic to orthochromatic erythroblasts. Dual staining of CD36 with Annexin V then provided a robust and high throughput compatible assay for monitoring IE, as illustrated in Figure 1I. A cell gate was used in order not to exclude potential apoptotic cells. Single cells were gated by FSC-H vs FSC-A. The vertical quadrant divider for the IE plots was determined using a CD36-Vioblue only stained negative control for Annexin V (AnV) positive cells (right hand plot). The horizontal quadrant divider was set at 10^3 the chosen cut-off, separating the CD36high and CD36low cell populations, allowing comparison of cell maturation within each experiment.

☒ Tick this box to confirm that a figure exemplifying the gating strategy is provided in the Supplementary Information.
